# Elevated temperatures do not trigger a conserved metabolic network response among thermotolerant yeasts

**DOI:** 10.1186/s12866-019-1453-3

**Published:** 2019-05-17

**Authors:** Mathias Lehnen, Birgitta E. Ebert, Lars M. Blank

**Affiliations:** iAMB – Institute of Applied Microbiology, ABBt – Aachen Biology and Biotechnology, RWTH Aachen University, Worringer Weg 1, D-52074 Aachen, Germany

**Keywords:** Thermotolerance, Quantitative physiology, ^13^C-metabolic flux analysis, *Kluyveromyces marxianus*, *Ogataea (Hansenula) polymorpha*, Metabolism

## Abstract

**Background:**

Thermotolerance is a highly desirable trait of microbial cell factories and has been the focus of extensive research. Yeast usually tolerate only a narrow temperature range and just two species, *Kluyveromyces marxianus* and *Ogataea polymorpha* have been described to grow at reasonable rates above 40 °C. However, the complex mechanisms of thermotolerance in yeast impede its full comprehension and the rare physiological data at elevated temperatures has so far not been matched with corresponding metabolic analyses.

**Results:**

To elaborate on the metabolic network response to increased fermentation temperatures of up to 49 °C, comprehensive physiological datasets of several *Kluyveromyces* and *Ogataea* strains were generated and used for ^13^C-metabolic flux analyses. While the maximum growth temperature was very similar in all investigated strains, the metabolic network response to elevated temperatures was not conserved among the different species. In fact, metabolic flux distributions were remarkably irresponsive to increasing temperatures in *O. polymorpha*, while the *K. marxianus* strains exhibited extensive flux rerouting at elevated temperatures.

**Conclusions:**

While a clear mechanism of thermotolerance is not deducible from the fluxome level alone, the generated data can be valued as a knowledge repository for using temperature to modulate the metabolic activity towards engineering goals.

**Electronic supplementary material:**

The online version of this article (10.1186/s12866-019-1453-3) contains supplementary material, which is available to authorized users.

## Background

For centuries, yeasts have been essential to many applications for the betterment of human life. With the rise of metabolic engineering and due to manifold beneficial traits, ranging from ethanol tolerance to protein glycosylation, these organisms have also found various applications outside the baking and beverage industries [1, 2]. While the engineering of microbial strains for biotechnological processes has profited intensively from rapid advances in the accuracy, applicability, and availability of genetic engineering tools, rational strain development must be based on comprehensive knowledge on genomics, physiology, and metabolism [3, 4]. Metabolic flux analysis provides the means for the necessary exploration of the intracellular metabolic network operation beyond measuring growth, substrate consumption, and metabolite secretion [5–7].

Thermotolerance is a rare trait among yeasts, but has been described for few species and was improved for example in baker’s yeast [8, 9]. Thermotolerant yeasts have been applied in industrial processes but not exclusively for the reason of thermotolerance [10, 11]. While a general interest in thermotolerant strains can be raised from universal process requirements, like less expensive cooling and lower risk of contamination, a popular process example that would benefit extensively from thermotolerant strains is the production of second-generation bioethanol [12]. *Kluyveromyces marxianus* (synonym: *Candida macedoniensis*) and *Ogataea polymorpha* (synonyms: *Hansenula polymorpha, Candida thermophila, Pichia angusta*) are the two most prominent examples of thermotolerant yeasts and both have beneficial traits besides their thermotolerance. *K. marxianus* can grow at staggering growth rates and produces ethanol if cultivated at temperatures above 40 °C [13]. *O. polymorpha* is methylotrophic and exhibits desirably low levels of hyperglycosylation [13, 14]. Conversion of xylose to ethanol at elevated temperatures has also been reported [15].

**Fig. 1:**
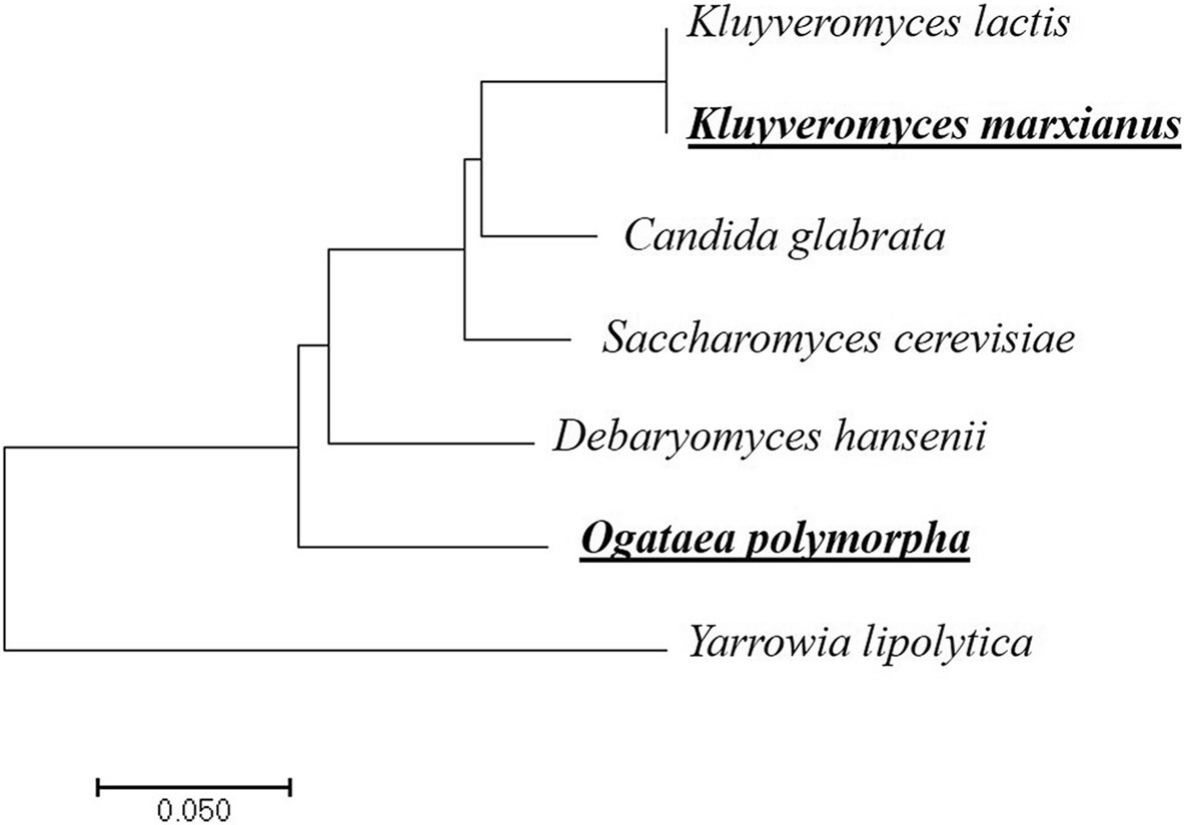
Phylogenetic tree of thermotolerant yeast and related species. The relationships were inferred using the Neighbor-Joining method [16]. The tree is drawn to scale based on evolutionary distances, which were calculated with MEGA7 [17] using the Maximum Composite Likelihood method [18] based on 26S ribosomal RNA sequences

The relationships of these taxa are shown in Fig. 1, contextualized with prominent yeast evolution events [19].

Both species seem to have surpassed their non-conventional status and have been successfully targeted with novel metabolic engineering tools [20–24]. However, the mechanisms enabling thermotolerance remain unclear and no quantitative physiology data including information on intracellular fluxes is available for growth at elevated temperatures. The aim of this study was to provide this missing data and explore trends in the metabolic network responses of the most prominent thermotolerant yeasts.

## Results

**Fig. 2:**
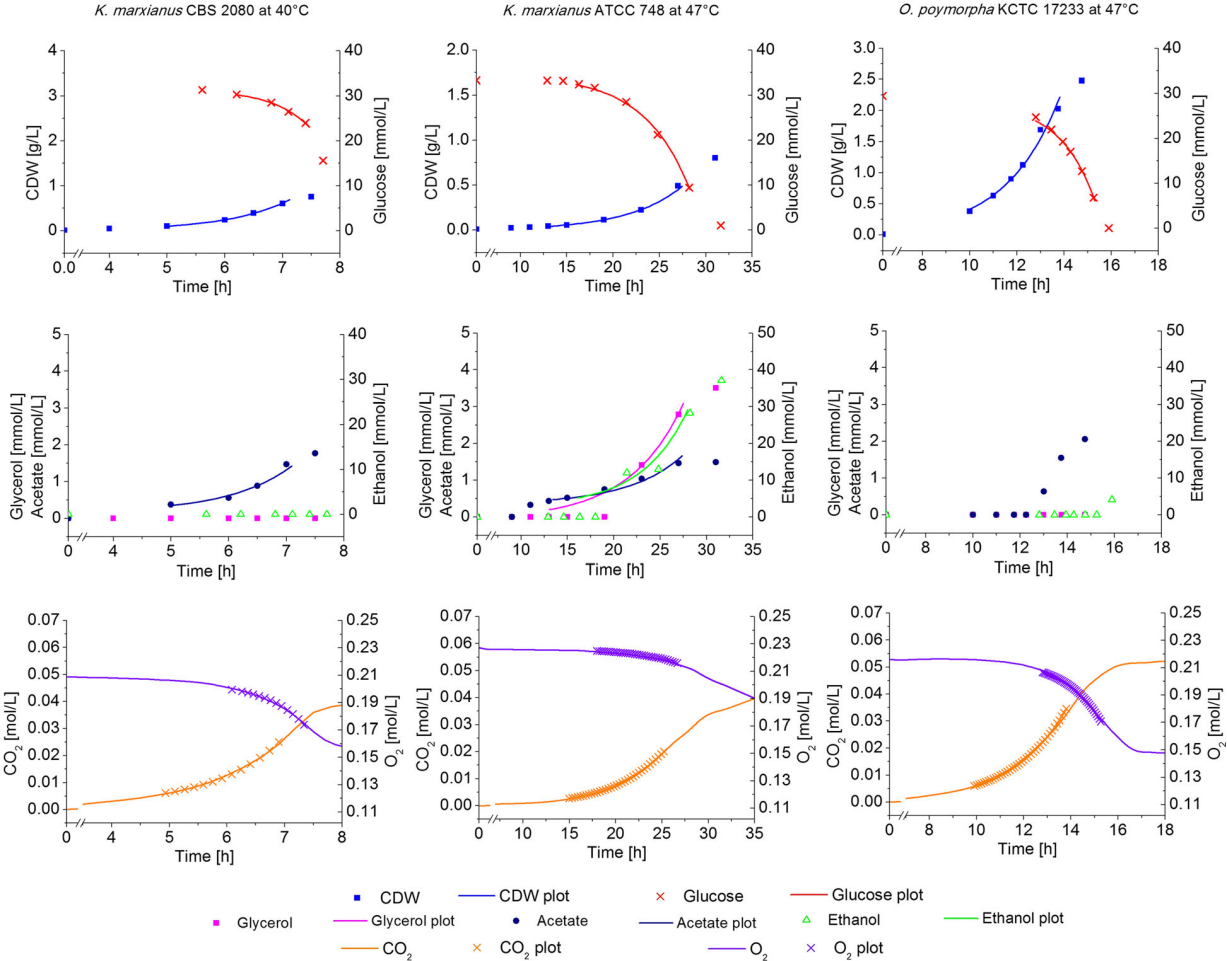
Experimental data, generated in minimal medium batch cultivations, and corresponding non-linear fits with (left column) - *K. marxianus* CBS 2080 at 40 °C, (middle column) – *K. marxianus* ATCC 748 at 47 °C, and (right column) – *O. polymorpha* KCTC 17233 at 47 °C. Equivalent data sets were generated for six strains in total and at temperatures varying from 30 °C to 49 °C. The physiological rates calculated from these data sets are condensed in Fig. 3 and furthermore given in more detail in Additional file 2: Tables S1 and S2

**Fig. 3:**
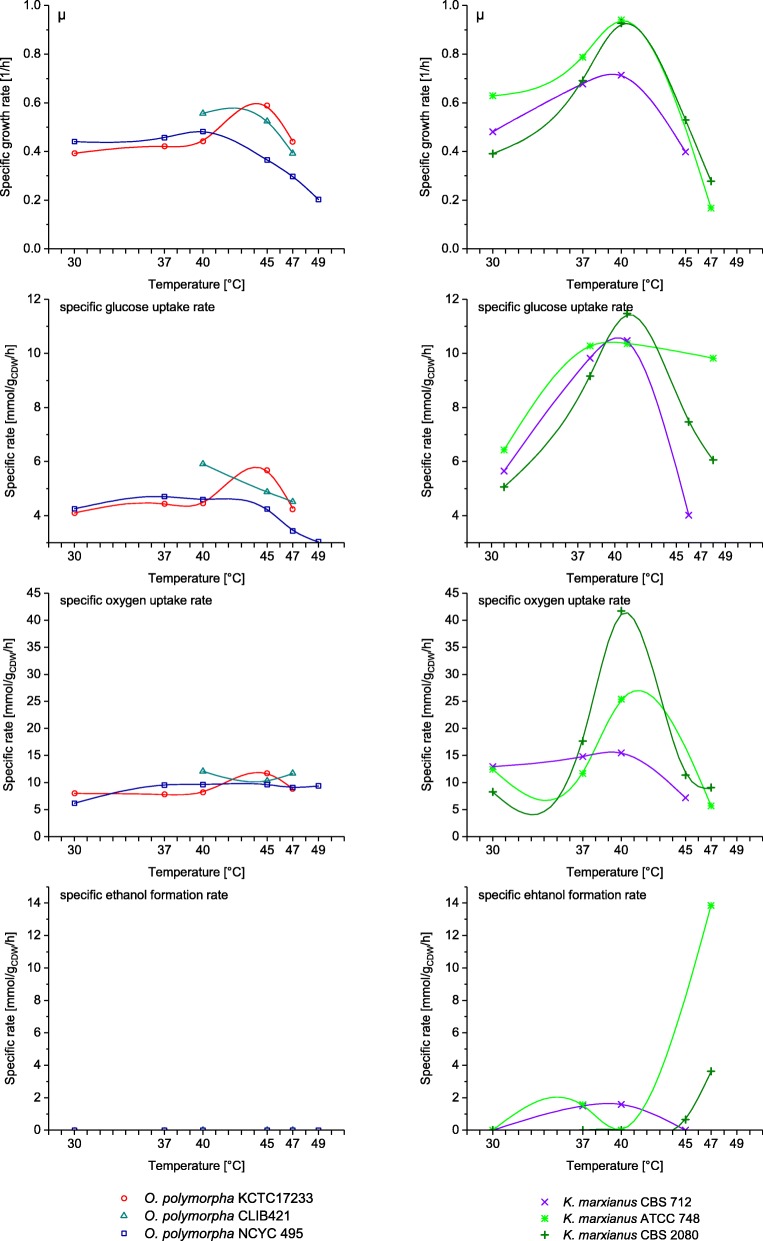
Physiological data of *O. polymorpha* strains (left hand side) and *K. marxianus* strains (right hand side) from batch experiments on glucose at different temperatures. Depending on the strain, the cultivation temperature was varied between 30 °C and 49 °C in independent experiments. To better illustrate the adaptive responses, the extracellular rates were accentuated with a line corresponding to a fitted spline curve generated from the measured data. The data of *O. polymorpha* CLIB 421 at 40 °C are taken from Lehnen et al. [26]. μ – specific growth rate

Available literature on thermotolerant yeasts clearly suggests that *K. marxianus* and *O. polymorpha* perform well at high temperatures [13, 25]. This was further supported by an initial screening of strains from an internal strain collection (data not shown). To obtain a comprehensive overview on physiology and flux distributions at elevated temperatures and to also explore differences between those two species, three strains of each of the two species were examined at temperatures ranging from 30 to 49 °C. Each of these experiments yielded a comprehensive physiological data set from which specific growth rates, substrate consumption and metabolite secretion rates were calculated. The physiological data sets of three growth experiments are exemplarily shown in Fig. 2); the entirety of calculated physiological data from the shake-flask batch cultures is summarized in Fig. 3. Secretion rates of acetate and glycerol were marginal in all experiments and are not shown here but are presented in the Additional file 1: Figure S1.

The *Ogataea* strains showed a phenotype that was highly stable over the investigated range of temperatures, especially up to 40 °C, with growth rates narrowing around 0.4 1/h, glucose uptake rates of 4 mmol/g_CDW_/h, and oxygen uptake rates of around 8 mmol/g_CDW_/h. No glycerol production was observed, and significant amounts of acetate were produced only by *O. polymorpha* KCTC 17233 at 37 °C and 40 °C. *O. polymorpha* KCTC 17233 was furthermore unique in having a maximum growth rate at 47 °C, while all other strains showed a maximum growth rate at 40 °C. Although *O. polymorpha* KCTC 17233 was still able to grow at 49 °C, this dataset is excluded here because growth was not exponential. The data of *O. polymorpha* CLIB 421 at 40 °C has been used in a previous study to validate a ^13^C-based metabolic flux analysis model [26] and is included here to complement the strain’s fluxome and physiological data at higher temperatures. This strain showed a similar physiological behavior as *O. polymorpha* NCYC 495 but did not produce any byproducts at the tested temperatures. As an example of the common *Ogataea* phenotype, a comprehensive flux map of *O. polymorpha* KCTC 17233 at 47 °C is given in Fig. 5.

**Fig. 4:**
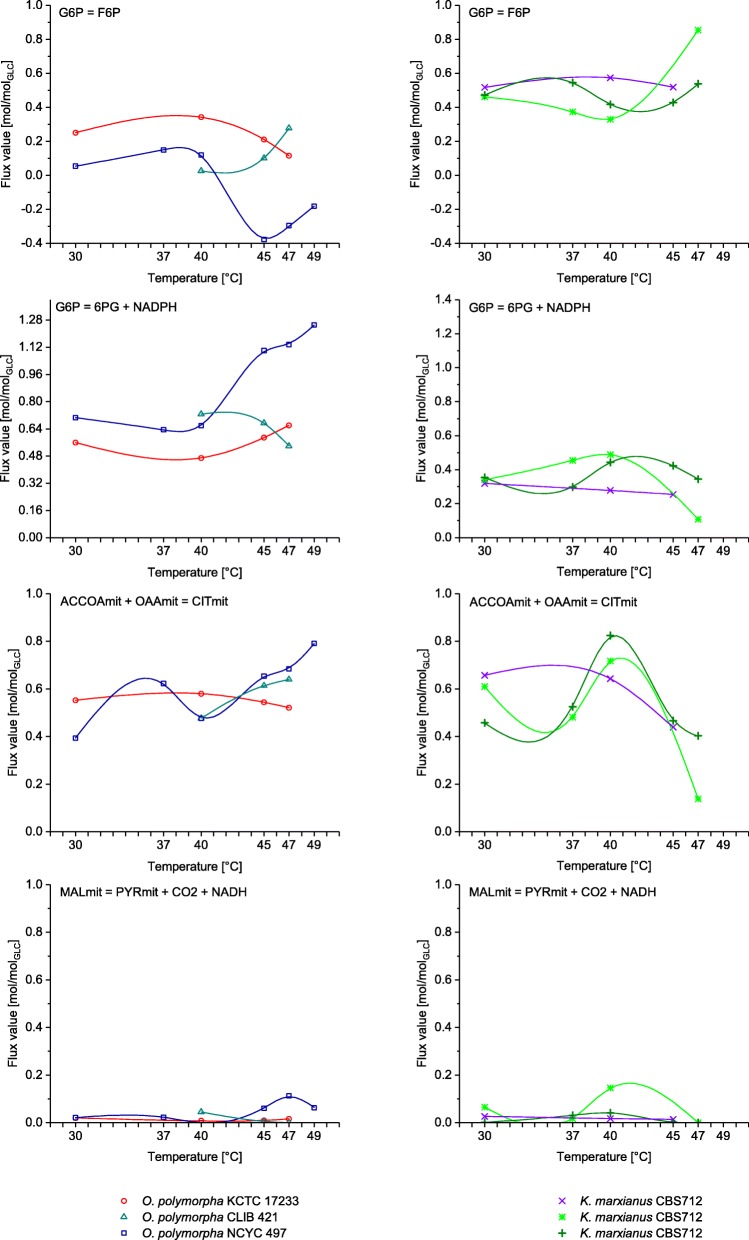
Excerpt of metabolic network responses of *O. polymorpha* strains (left hand side) and *K. marxianus strains* (right hand side) in glycolysis (represented by the the phosphoglucose isomerase reaction), the pentose phosphate pathway (represented by the glucose-6-phosphate dehydrogenase reaction), the tricarboxylic acid cycle (represented by the citrate synthase reaction), and malic enzyme activity to cultivation at higher temperatures. The malic enzyme fluxes producing NADH and NADPH were combined in this representation. Flux values are normalized to the glucose uptake rate. To better illustrate adaptive responses, the fluxes are accentuated with a spline curve fitted to the computed flux data. The reactions are designated by the stoichiometric reaction equation as defined in the model. G6P – glucose-6-phosphate; F6P – fructose-6-phosphate; 6PG – 6-phosphogluconate; ACCOA – acetyl-CoA; OAA – oxaloacetate CIT – citrate; MAL – malate; PYR – pyruvate; mit –mitochondrial localization

In contrast to the phenotypic stability of the *Ogataea* strains, the *Kluyveromyces* strains were highly adaptive to temperature changes exhibiting a variety of phenotypes with large differences also between the individual strains. Two outstanding phenotypes, one defined by a very high growth rate and a second, defined by a high ethanol yield, have already been subject of extensive research [13, 27] and were observed in *K. marxianus* strains at 40 °C and 47 °C, respectively. Two strains, specifically *K. marxianus* CBS 712 and *K. marxianus* ATCC 748, showed growth rates above 0.9 1/h at 40 °C, which is approximately double the maximal growth rate reported for *S. cerevisiae* [28]. These maximum growth rates were accompanied by high glucose (up to 12 mmol/g_CDW_/h) and oxygen uptake rates (40 mmol/g_CDW_/h), and TCA cycle rates reaching 9.5 mmol/g_CDW_/h (Figs. 3 and 4). Astonishingly, these high growth rates were achieved without compromising the biomass yield, which was maintained above 0.5 g_CDW_/g_glucose_. At even higher temperatures, two of the *K. marxianus* strains produced ethanol reaching production rates as high as 14 mmol/g_CDW_/h in *K. marxianus* ATCC 748 at 47 °C. The entire collection of the obtained physiological data is given in the Additional file 2: Tables S1 and S2.

**Fig. 5:**
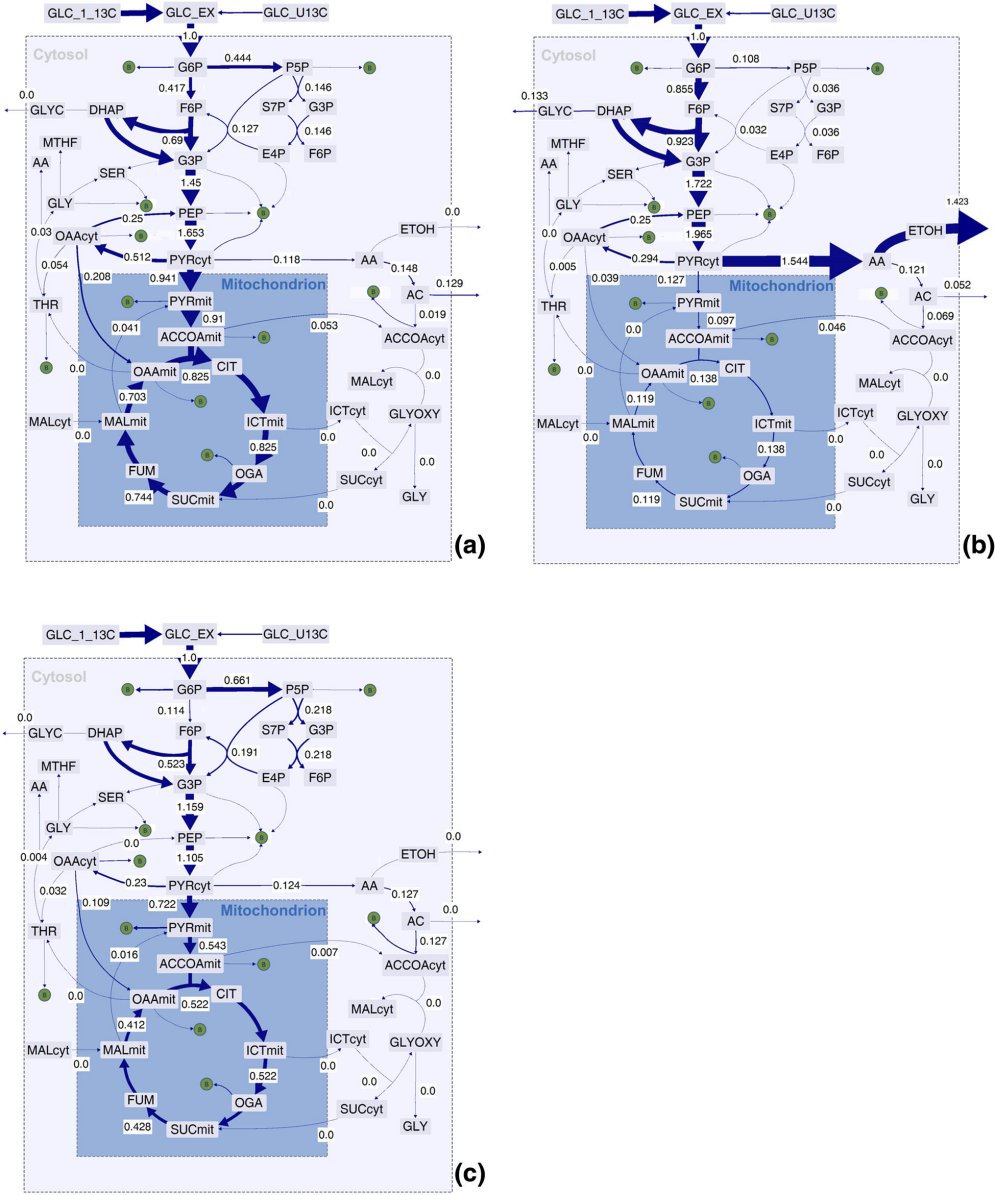
Simulated flux distributions of (**a**) - *K. marxianus* CBS 2080 during growth at 40 °C, (**b**) – *K. marxianus* ATCC 748 during growth at 47 °C, and (**c**) – *O. polymorpha* KCTC 17233 during growth at 47 °C. The boxed numbers next to the reaction arrows represent the flux values, which were normalized to the glucose uptake rate. Arrow thickness was scaled to the flux value for enhanced visualization. Metabolite abbreviations are defined in the Additional file 3: Flux solutions & sensitivity

To get a better insight into how intracellular flux changes contribute to the observed phenotypic changes, we conducted ^13^C-metabolic flux analyses (^13^C-MFA) for all strains and for all temperatures. Both genera showed distinctive flux distributions characterized by a very high pentose phosphate pathway (PPP) flux in the *Ogataea* strains and comparably higher glycolytic and TCA cycle fluxes for *Kluyveromyces*. Compared with the Crabtree-positive yeast *S. cerevisiae* at 30 °C under otherwise identical culture conditions, these oxidative metabolic schemes sustained much higher rates of redox cofactor reduction in all experiments (Figs. 4 and 5) [26].

To simulate the flux distributions of *O. polymorpha* NCYC 495 above 40 °C, an additional NADPH consuming flux had to be allowed to generate flux solutions, which were statistically acceptable as checked by a χ^2^ test. For the datasets generated at temperatures of 45 °C and above this relaxation of the NADPH balance resulted in flux solutions with a partially cyclic PPP activity, i.e., relative PPP fluxes above 1 mol/mol_glucose_.

Not surprisingly, the TCA cycle activity of the investigated Crabtree-negative yeasts was considerably higher in all strains and at all investigated temperatures compared to *S. cerevisiae* under standard growth conditions with a high of 80% (mol/mol_glucose_) [26, 29]. However, at temperatures above 40 °C, the TCA cycle activity showed a stark decrease accompanied with ethanol formation in two of the *Kluyveromyces* strains (Fig. 3), while such a metabolic change was not observed with any of the tested *O. polymorpha* strains.

An excerpt of the flux distribution data of all investigated strains is presented in Fig. 4 focusing on important central metabolic reactions. A collection of all generated flux distribution data and flux maps is given in Additional file 3: Flux solutions & sensitivity.

## Discussion

The most notable difference in the metabolic response of *Ogataea* and *Kluyveromyces* strains to increased cultivation temperature was the remarkable metabolic variability of *Kluyveromyces* strains, which is absent in *Ogataea*. Special focus of the discussion around the metabolic basis of thermotolerance lies on the activity of the PPP, which was increased in all investigated strains compared to *S. cerevisiae* [28], but it still clustered on distinct levels in the different species. The divergence of metabolic responses becomes readily apparent from the observed secretion rates of metabolites. While the secretion of glycerol, acetate, and ethanol increases in correlation with temperature in *S. cerevisiae* [30], such a correlation was not observed for the thermotolerant species. Although cells may profit from the thermoprotective property of glycerol [30], none of it was produced by *Ogataea* and no correlation with temperature was found for *Kluyveromyces*. Secretion of pyruvate, as described by Fonseca et al. [31] for a different *K. marxianus* strain and during growth on higher sugar concentrations, was not observed in the present study. This indicates that the response to increased temperature is not conserved among thermotolerant yeast species, much less among yeasts in general.

In the *Kluyveromyces* strains, the temperature increase resulted in a stark increase of metabolic activities, thereby being in accordance with the temperature dependence of reaction rates described by Arrhenius. The optimal temperature of all three *Kluyveromyces* strains was around 40 °C at which they showed approximately doubled glucose uptake and growth rates compared to growth at 30 °C. Apparently, at mesophilic temperatures, the strain is not exploiting its maximal metabolic capacity. Additionally, we observed an up to 2-fold increase of the TCA cycle activity indicating that the elevated temperatures cause a typical metabolic stress response. Specifically, the cells experience an increased mitochondrial membrane permeability, concomitant proton influx, protein damage, and lipid peroxidation, which needs to be counteracted by ATPase activity and cell repair mechanisms [32]. These mechanisms explain an increased ATP demand and observed high flux through the TCA cycle [33], climaxing at 40 °C in *Kluyveromyces* (Fig. 3). At temperatures beyond 40 °C, the collapse of the oxygen uptake rate (Fig. 3) and the TCA cycle activity (Fig. 4) indicates that the damage to the mitochondrial membranes reaches a level that can no longer support respiratory metabolism as previously discussed by M Zhang, J Shi and L Jiang [34]. Alternatively, *K. marxianus* might actively downregulate the genes of the respiratory chain as has been observed for another strain of this species [35] and discussed as a means to reduce the formation of reactive oxygen species. Both mechanism can explain the onset of fermentative metabolism, apparent from the production of ethanol and acetate.

In contrast to *Kluyveromyces*, no drastic metabolic shifts, but relatively moderate changes in the flux distributions in *Ogataea* strains were observed in accordance with the relatively stable phenotype. Of the three tested strains, only *O. polymorpha* NCYC 495 showed a drop in the glycolytic flux for growth at or above 45 °C and an associated increase in PPP and TCA cycle activity. This might be an indication that thermotolerance in *Ogataea* is mainly governed by structural predisposition, for example membrane composition that allows for higher stability, or protective mechanisms like glutathione reductase.

One common trait in all flux distributions was the elevated PPP activity compared with *S. cerevisiae* grown at 30 °C and under standard cultivation conditions. This might be correlated to the high NADPH demand under increased temperatures for glutathione reductase-mediated protection against oxidative stress [36–38]. The rerouting of metabolic flux from glycolysis to PPP is reported to be a conserved response to oxidative stress [39] and has also been observed in *S. cerevisiae* [30, 40]. *Ogataea* flux distributions were further characterized by a high PPP flux even at moderate temperatures. In this methylotrophic yeast, high PPP activity may also be an inherent characteristic as several PPP associated enzymes are involved in the pathway for methanol assimilation [41]. A cyclic mode of the PPP, as simulated for *O. polymorpha* NCYC 495 above 40 °C, has recently been discussed as a means to sustain increased NADPH demands [42]. The corresponding, massive increase of excess NADPH production further supports glutathione mediated thermotolerance of this strain [37, 38]. Likewise, cyclic operation of the Entner-Doudoroff pathway has been determined for obligate aerobic bacteria, which might face an increased NADPH demand due to reactive oxygen species and other environmental stresses [43–45].

However, a higher potential for glutathione mediated protection against oxidative stress as it results from higher NADPH production is not sufficient for thermotolerance. While it is known that *S. cerevisiae*, too, is capable of achieving very high PPP activities [40, 46], this strain is not capable of growing at elevated temperatures and has been shown by Blank and Sauer [28] to maintain a low PPP activity in batch at 37 °C [28]. The high PPP activity reported for thermosensitive *P. pastoris* [47] and *K. lactis* [48] further support this hypothesis. Hence, structural specialties of the membranes and enzymes of thermotolerant yeasts seem to be more important conditions for metabolic activity at high temperatures [49, 50].

From an applied perspective, the generated quantitative data is valuable for strain and process development purposes as it demonstrates how temperature modulates metabolic fluxes and can be used to explore and tune desirable phenotypes. The presented, comprehensive overview of phenotypes and flux distributions of several interesting *Kluyveromyces* and *Ogataea* strains forms a foundation for a reasonable choice of a suitable production host with desirable properties. For example, strains with high PPP activities have an industrial potential for the production of aromatic compounds, relying on the PPP intermediate erythrose-4-phosphate [51]. In *K. marxianus*, this positive trait is further enhanced by its fast growth rate of 0.94 h^− 1^ and the simultaneous high biomass yield of 0.5 g_CDW_/g_glucose_. Temperature-insensitive physiology, another central result of this study, is highly desirable to confer robustness towards temperature gradients often encountered in large-scale and solid-state fermentations [52, 53].

## Conclusion

The generated physiology and fluxome data suggests that there are no highly conserved metabolic traits among thermotolerant yeasts. While the physiological changes and observed metabolic shifts indicate a response to several of the most prominent effects of high temperature and concomitant stresses, like loss of membrane integrity and protective action against reactive oxygen species [34], the response behavior of the various strains varied significantly. When compared to the metabolic fluxes of *S. cerevisiae*, both analyzed species show increased PPP and TCA cycle activity under all temperature conditions, paired with high biomass yields even at very high growth rates. These quantitative physiological and fluxome data underpin the potential of these two species for biomanufacturing purposes.

## Materials and methods

### Strains and culture conditions

The strains used in this study were *Kluyveromyces marxianus* ATCC 748, *Kluyveromyces marxianus* CBS 2080, *Kluyveromyces marxianus* CBS 712, *Ogataea polymorpha* CLIB 421, *Ogataea polymorpha* NCYC 495, and *Ogataea polymorpha* KCTC 17233. To produce high quality flux distributions, physiological data, which was used to constrain the flux estimations, and samples of ^13^C-labeled biomass were obtained from growth experiments, executed according to a standard protocol, only changing strain or temperature between experiments. Cultivations were performed in 1.3 L shake flasks, equipped with O_2_- and CO_2_-sensors (BlueSens gas sensor GmbH, Herten, Germany). The flasks were filled with 50 mL of Verduyn medium [54], and shaken at 130 rpm with an amplitude of 30 mm as described in Lehnen et al. [26]. The closed shake flask was equipped with an air-tight sample port. The medium contained per liter 5 g (NH_4_)_2_SO_4_, 3 g KH_2_PO_4_, 0.5 g MgSO_4_∙7H_2_O, 4.5 mg ZnSO_4_∙7H_2_O, 0.3 mg CoCl_2_∙6H_2_O, 1.0 mg MnCl_2_∙4H_2_O, 0.3 mg CuSO_4_∙5H_2_O, 4.5 mg CaCl_2_∙2H_2_O, 3.0 mg FeSO_4_∙7H_2_O, 0.4 mg NaMoO_4_∙2H_2_O, 1.0 mg H_3_BO_3_, 0.1 g KI, 15 mg EDTA, 0.05 mg biotin, 1.0 mg calcium pantothenate, 1.0 mg nicotinic acid, 25 mg inositol, 1.0 mg pyridoxine, 0.2 mg p-aminobenzoic acid, 1.0 mg thiamine. The medium was supplemented with 5 g/L glucose consisting of 80% (n/n) 1-^13^C-glucose and 20% (n/n) U-^13^C-glucose (both purchased from Sigma-Aldrich, Steinheim, Germany, with 99 atom-% purity), because it allows the determination of intracellular fluxes with good resolution at reasonable costs per experiment [7]. Precultures were grown in the same minimal medium but supplemented with naturally labeled glucose. Cells were harvested by centrifugation, washed with 0.9% NaCl solution, and used to inoculate the main cultures to a starting optical density (OD_600_) of 0.05. Biomass samples for determination of the ^13^C-labeling of proteinogenic amino acids by GC-MS analysis were taken during the exponential growth phase no earlier than five generations after inoculation.

### Physiological data acquisition and processing

During the exponential growth phase, samples were taken via the sample port to not disturb the gas composition in the head space as described by Lehnen et al. [26]. The OD_600_ was measured immediately after sampling. The samples were centrifuged at 13,000 × g for 5 min in a Thermo Scientific Heraeus Pico 17 tabletop centrifuge. The biomass pellet and the supernatant were stored separately at − 20 °C until further analysis. The pellet was used for GC-MS analysis of proteinogenic amino acids (see below). The concentrations of glucose and the excreted metabolites ethanol, glycerol, and acetate were determined by HPLC analysis of the supernatant. A constant flow of 0.8 mL/min of 5 mM H2SO4 was used to separate the metabolites on an Aminex HPX-87H column (Bio-Rad, Hercules, CA, USA) at 60 °C. Glucose, ethanol, and glycerol were quantified with a Shodex RI-101 detector, and a variable wavelength detector of an UltiMate 3000 HPLC system (Dionex, Sunnyvale, CA, USA) was used at 210 nm to quantify acetate. The cell dry weight (CDW) of samples was calculated from the measured OD_600_ and calibration curves, which were specific for the experimental conditions. The off-gas analysis system provided by BlueSens gas sensor GmbH (Herten, Germany) and included BCP-O_2_ and BCP-CO_2_ sensors connected to a BACCom12 communication box to obtain signlas in units Vol-% using the FermVis software. These signlas were transformed into molar concentrations as described by J Heyland, J Fu and LM Blank [29]. Exponential growth rate, substrate uptake rates, and metabolite secretion rates were calculated by a simultaneous nonlinear fit of the time-dependent concentration changes, using SigmaPlot Version 12.5 (Systat Software, Inc., San Jose, CA, USA) and the CO_2_ production rate was corrected for the insensitivity of the sensors towards labeled CO_2_, as described by M Lehnen, BE Ebert and LM Blank [26].

### ^13^C-labeling analysis

^13^C-labeling patterns of proteinogenic amino acids were determined from biomass samples taken in the mid-exponential growth phase. Approximately 0.3 mg of biomass was hydrolyzed at 105 °C for 6 h after resuspension of the pellet in 6 M HCl and then dried overnight at 80 °C. Derivatization of the dried hydrolysate was done in a mix of 30 μL acetonitrile and 30 μL N-methyl-N-tert-butyldimethylsilyl-trifluoroacetamide (CS-Chromatographie Service GmbH, Langerwehe, Germany) for one our at 85 °C for 1 h to allow derivatization. The derivatized samples were analyzed on a gas chromatography-mass spectrometry (GC-MS) triple quadrupole system. The GC-MS system consisted of a TRACE™ GC Ultra and a TSQ triple quadrupole MS with electron impact ionization (Thermo Fisher Scientific, Waltham, MA, USA) equipped with a TraceGOLD TG-5SilMS fused silica column (length, 15 m; inner diameter, 0.25 mm; film thickness, 0.25 μm) and was operated as described by Schmitz et al. [55]. Helium was used as carrier gas at a constant gas flow rate of 1 mL/min and at a split ratio of 1:15. The injector temperature was set to 270 °C, and the column oven was heated according to a ramped program. The initial temperature of 140 °C was held for 1 min, the temperature was then increased with a rate of 10 °C/min to a final value of 310 °C, which was held time for 1 min. iMS2Flux [56] was used to correct the raw GC-MS data for unlabeled biomass, introduced with the inoculum, and the natural abundance of heavy isotopes and to transform the corrected data into mass distribution vectors.

### ^13^C-MFA method and model constraints

To compute the intracellular fluxes, OpenFLUX was used [5, 57], based on a model that described the central carbon metabolism compartmented into cytosol and mitochondria [26] and included glycolysis, the pentose phosphate pathway (PPP), and the tricarboxylic acid (TCA) cycle, with approximately 50 reactions. Biomass production was linked to twelve intermediates of this central network [58]. The model was constrained with experimentally determined uptake and secretion rates, precursor drains into biomass, and compartmental localization of amino acid biosynthesis. As shown by M Lehnen, BE Ebert and LM Blank [26], it is most effective to constrain amino acid biosynthesis only for those amino acids where pathway localization information is unambiguous and to allow parallel biosynthesis in both compartments for all other amino acids. Based on the genome sequence of *O. polymorpha* NCYC 495, available from the homepage of the Joint Genome Institute (JGI) (http://genome.jgi.doe.gov/Hanpo2/Hanpo2.home.html, accessed in April 2016) and the genome sequence of *K. marxianus* KCTC 17555, which is deposited at DDBJ/EMBL/GenBank under the accession number AKFM00000000 [59], pathway localizations where computationally predicted using TARGETP 1.1 [60] and WoLF PSORT [61] for all strains as discussed in [62]. Detailed information on model constraints applied to evaluate the *Ogataea* and *Kluyveromyces* datasets, respectively, is given in the supplement.

## Additional files

Additional file 1: **Figure S1.** Supplementary physiological data of *K. marxianus* strains and *O. polymorpha* strains from batch experiments on glucose at different temperatures. **Figures S2-S25.** Flux maps of all strains for all tested temperatures. (DOCX 6050 kb)

Additional file 2: **Table S1.** Physiological rates of *K. marxianus* strains from batch experiments on glucose at different temperatures. Depending on the strain, the cultivation temperature was varied between 30 °C and 49 °C in different experiments. **Table S2.** Physiological rates of *O. polymorpha* strains from batch experiments on glucose at different temperatures. Depending on the strain, the cultivation temperature was varied between 30 °C and 49 °C in different experiments. (DOCX 26 kb)

Additional file 3: Optimal flux solutions for all simulated data sets, including the corresponding smallest sum of residual errors (fval) and the constraints for uptake and production rates of metabolites and biomass constituents, which were used in the simulations. (XLSX 516 kb)

## Abbreviations

CDW: Cell dry weight
GC-MS: Gas chromatography-mass spectrometry
MFA: Metabolic flux analysis
PPP: Pentose phosphate pathway
TCA: Tricarboxylic acid
