# Supplementary material for: Elevated temperatures do not trigger a conserved metabolic network response among thermotolerant yeasts
Source: BMC Microbiol. 2019 May 17;19:100. doi: 10.1186/s12866-019-1453-3 (PMC6525440; doi:10.1186/s12866-019-1453-3)
Supplement: Supplementary file 1 — Figure S1. Supplementary physiological data of K. marxianus strains and O. polymorpha strains from batch experiments on glucose at different temperatures. Figures S2-S25. Flux maps of all strains for all tested temperatures. (DOCX 6050 kb) [file 12866_2019_1453_MOESM1_ESM.docx]

**Additional file**


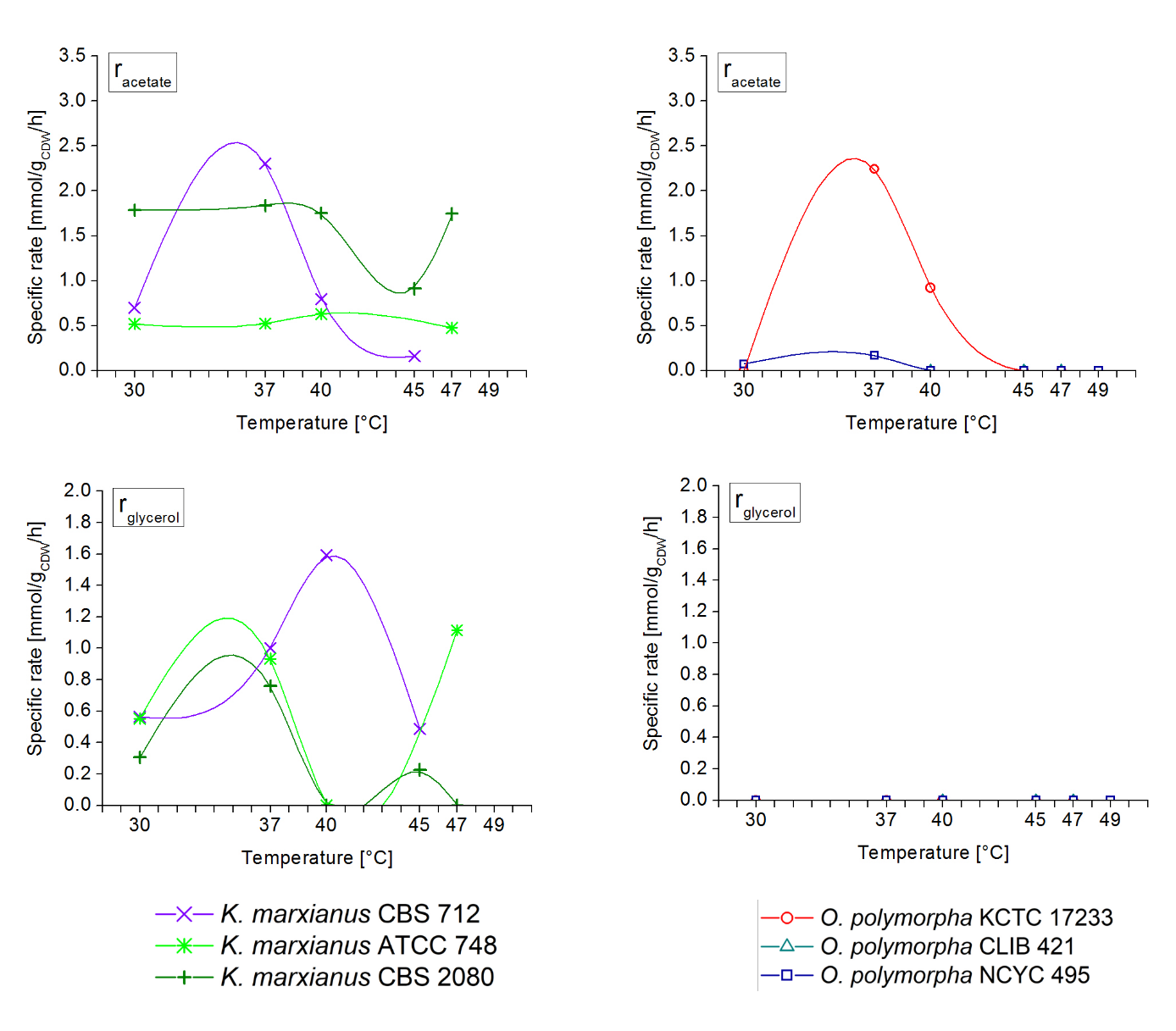


Additional file 1: Figure S1. Supplementary physiological data of K. marxianus strains (left hand side) and O. polymorpha strains (right hand side) from batch experiments on glucose at different temperatures. Depending on the strain, the cultivation temperature was varied between 30 °C and 49 °C in different experiments. To better illustrate adaptive responses of extracellular rates, the rates were accentuated with a line corresponding to a fitted spline curve generated from the measured data. The data of O. polymorpha CLIB 421 at 40°C are taken from (Lehnen et al., 2017). r_acetate_ – specific acetate production rate; r_glycerol_ – specific glycerol production rate.


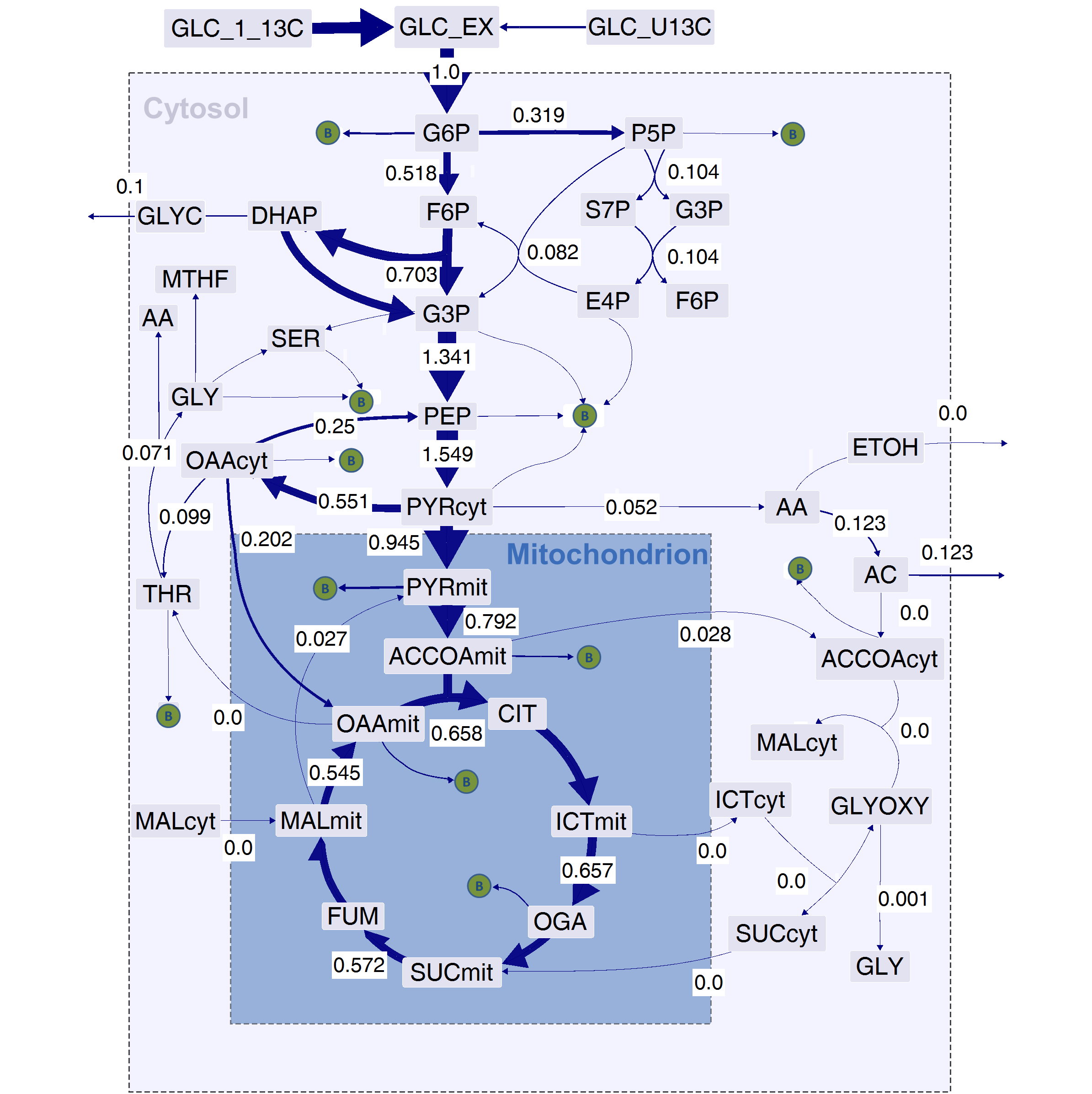


Additional file 1: Figure S2. Simulated flux distributions of K. marxianus CBS 712 during exponential growth at 30 °C The boxed numbers next to reaction arrows represent the flux values, which were normalized to the glucose uptake rate. Arrow thickness was scaled to the flux value for enhanced visualization.


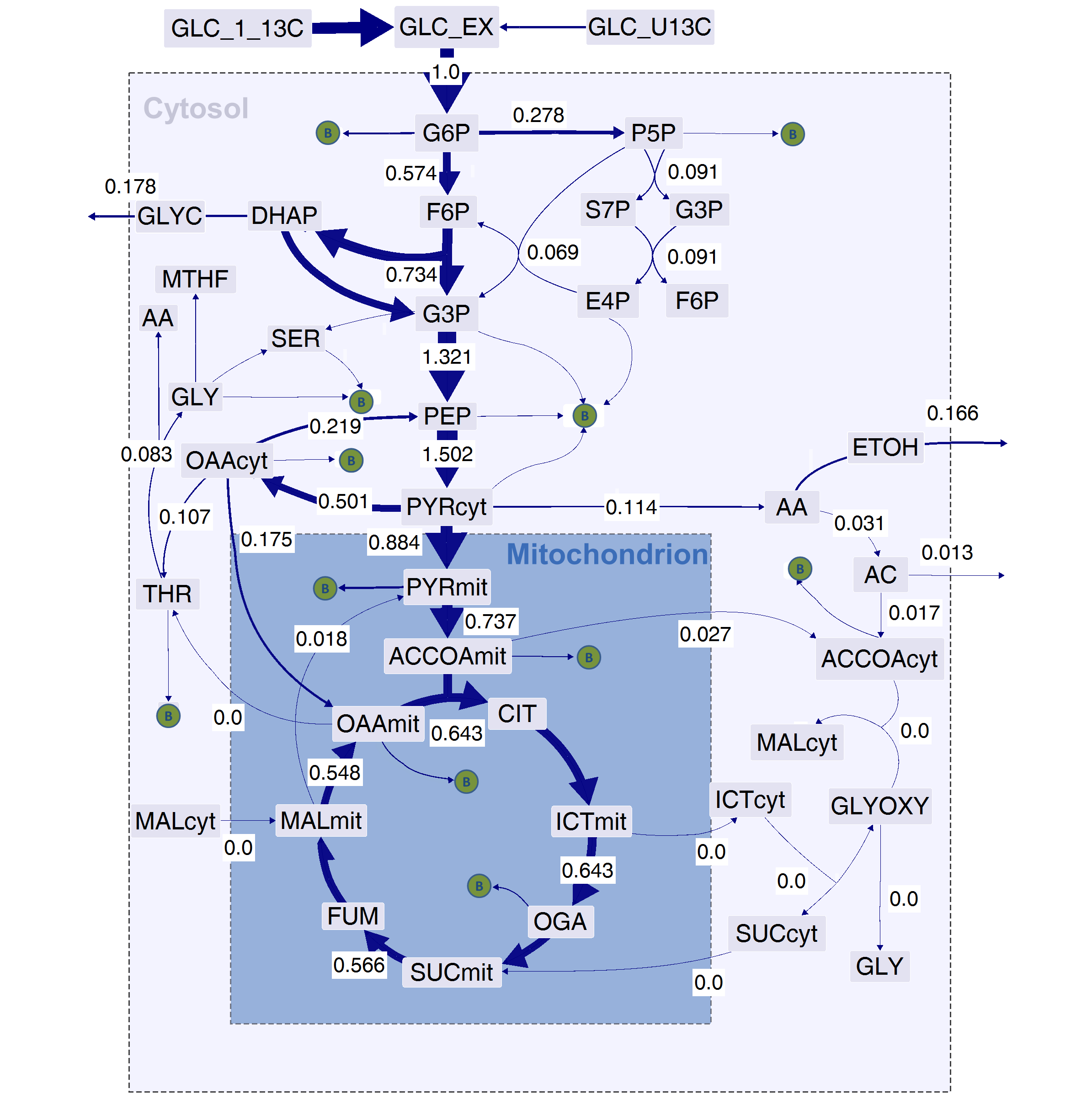


Additional file 1: Figure S3. Simulated flux distributions of K. marxianus CBS 712 during exponential growth at 40 °C The boxed numbers next to reaction arrows represent the flux values, which were normalized to the glucose uptake rate. Arrow thickness was scaled to the flux value for enhanced visualization.


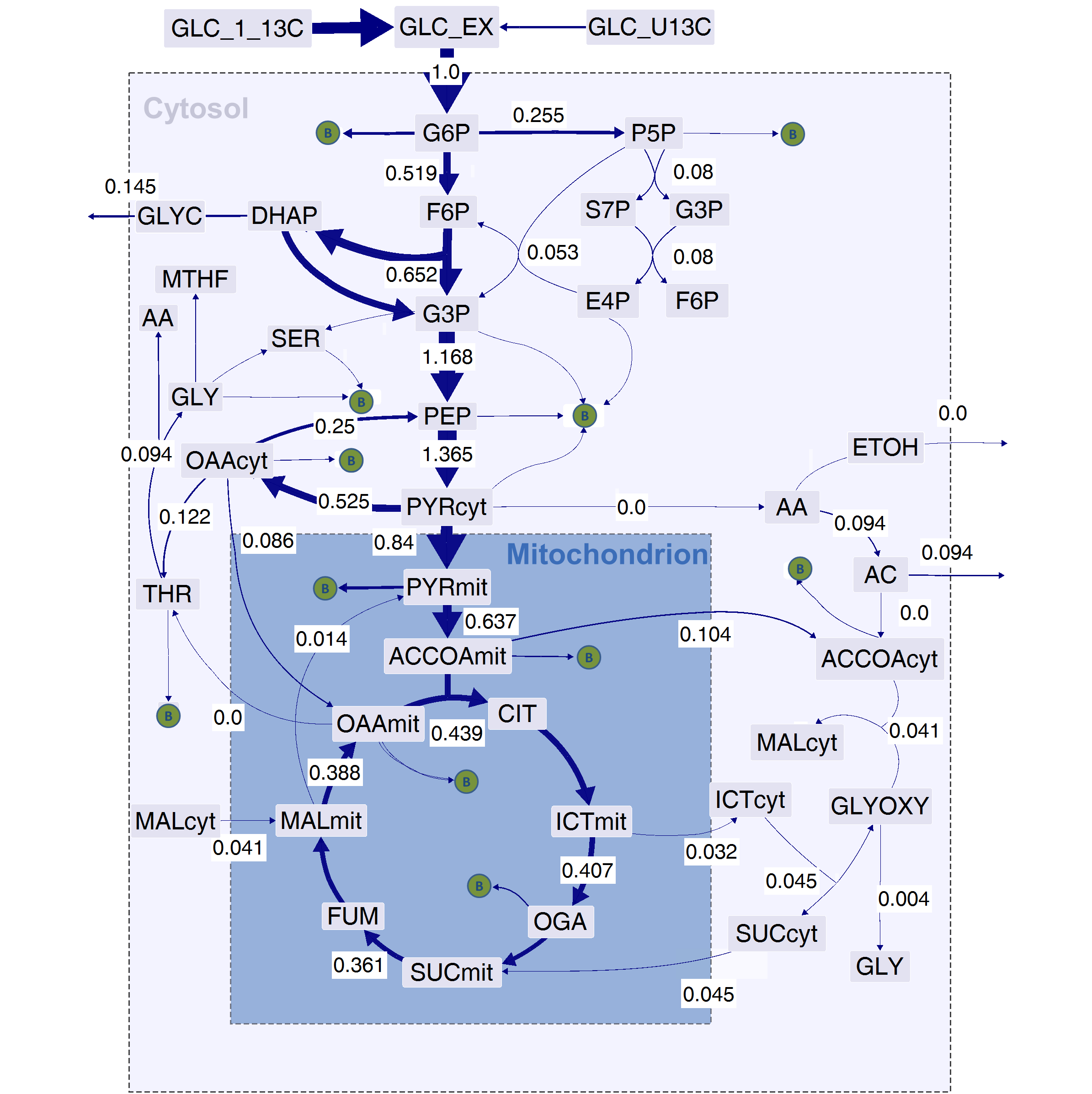


Additional file 1: Figure S4. Simulated flux distributions of K. marxianus CBS 712 during exponential growth at 45 °C The boxed numbers next to reaction arrows represent the flux values, which were normalized to the glucose uptake rate. Arrow thickness was scaled to the flux value for enhanced visualization.


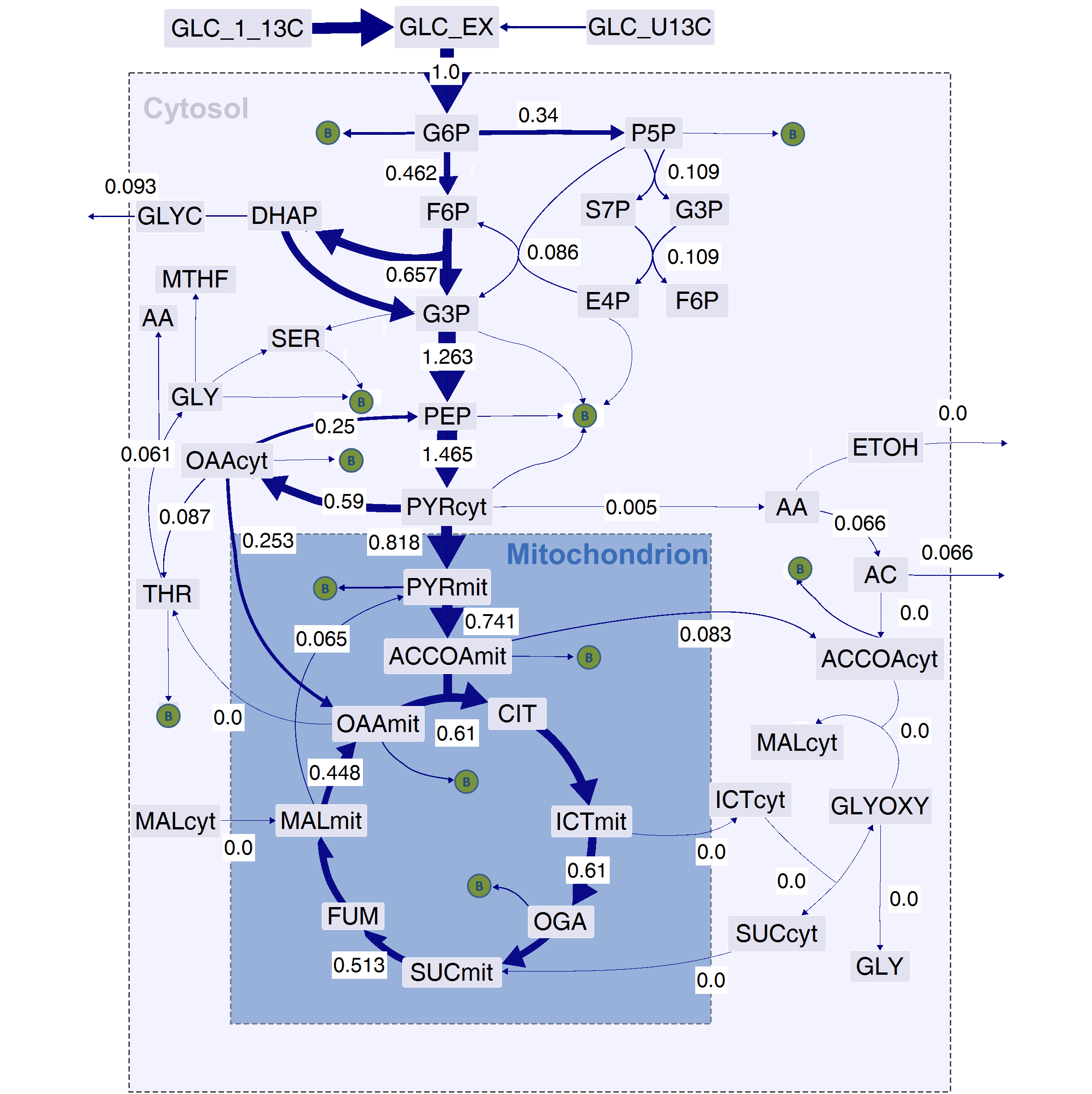


Additional file 1: Figure S5. Simulated flux distributions of K. marxianus ATCC 748 during exponential growth at 30 °C The boxed numbers next to reaction arrows represent the flux values, which were normalized to the glucose uptake rate. Arrow thickness was scaled to the flux value for enhanced visualization.


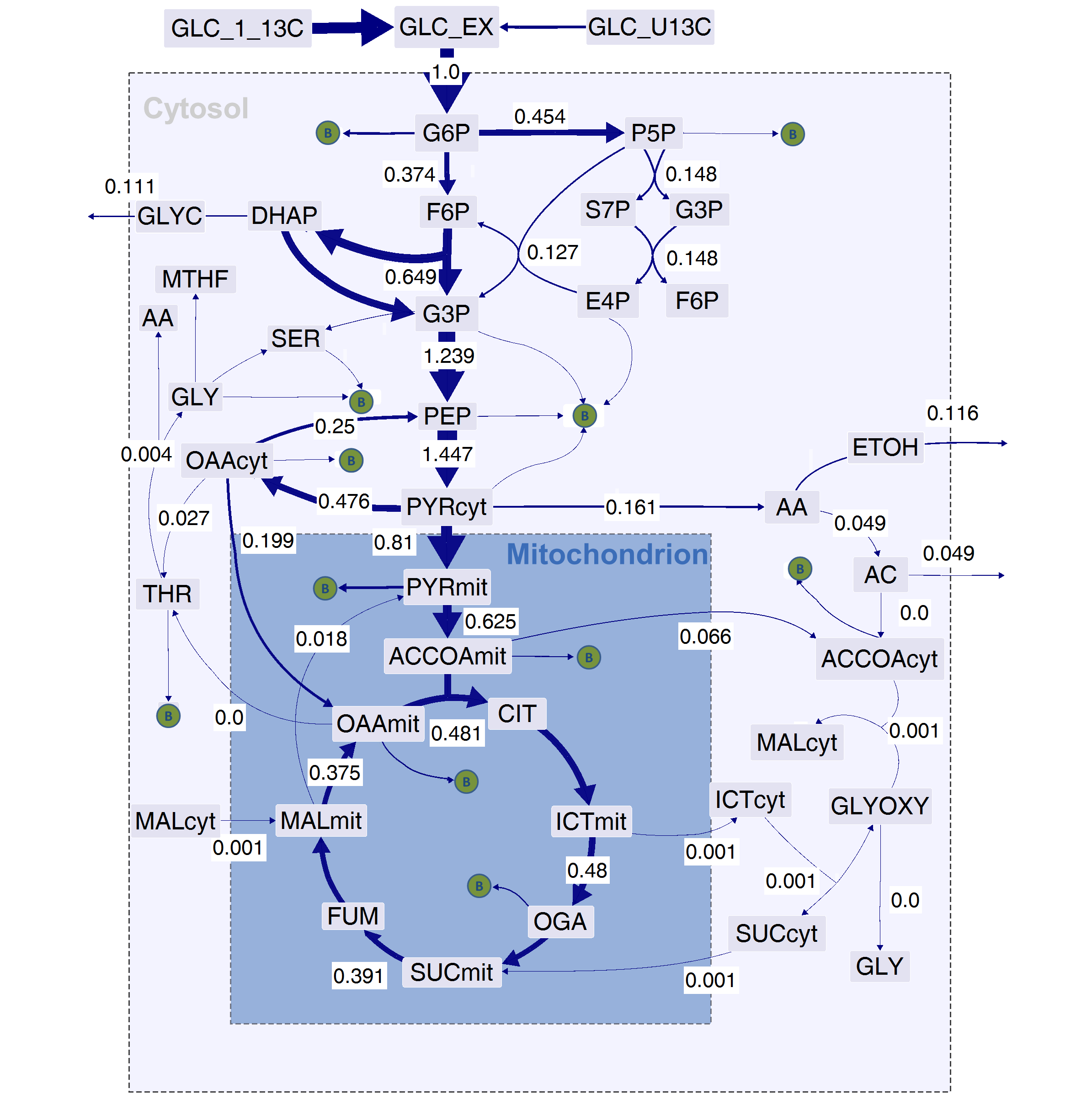


Additional file 1: Figure S6. Simulated flux distributions of K. marxianus ATCC 748 during exponential growth at 37 °C The boxed numbers next to reaction arrows represent the flux values, which were normalized to the glucose uptake rate. Arrow thickness was scaled to the flux value for enhanced visualization.


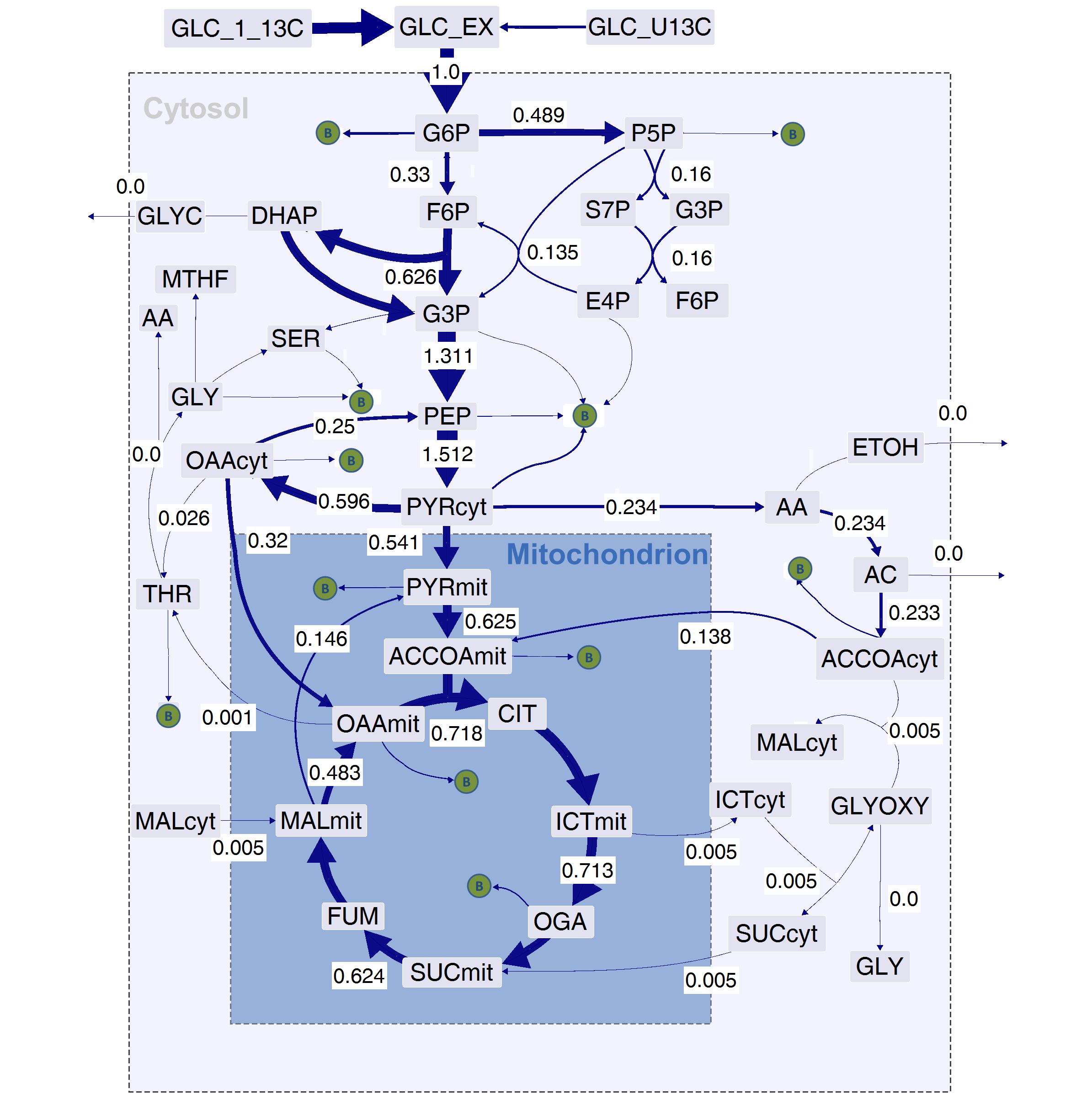


Additional file 1: Figure S7. Simulated flux distributions of K. marxianus ATCC 748 during exponential growth at 40 °C The boxed numbers next to reaction arrows represent the flux values, which were normalized to the glucose uptake rate. Arrow thickness was scaled to the flux value for enhanced visualization.


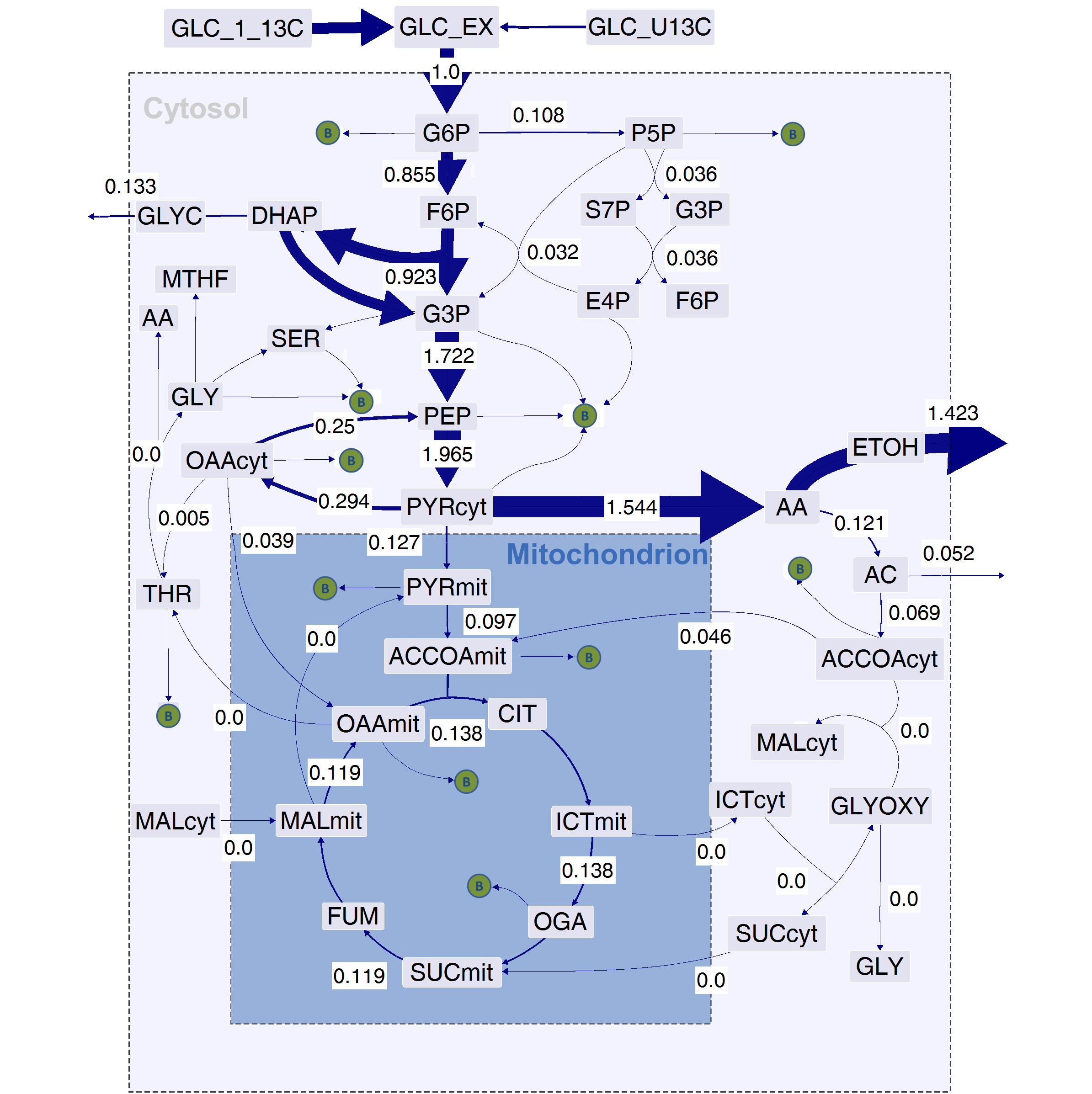


Additional file 1: Figure S8. Simulated flux distributions of K. marxianus ATCC 748 during exponential growth at 47 °C The boxed numbers next to reaction arrows represent the flux values, which were normalized to the glucose uptake rate. Arrow thickness was scaled to the flux value for enhanced visualization.


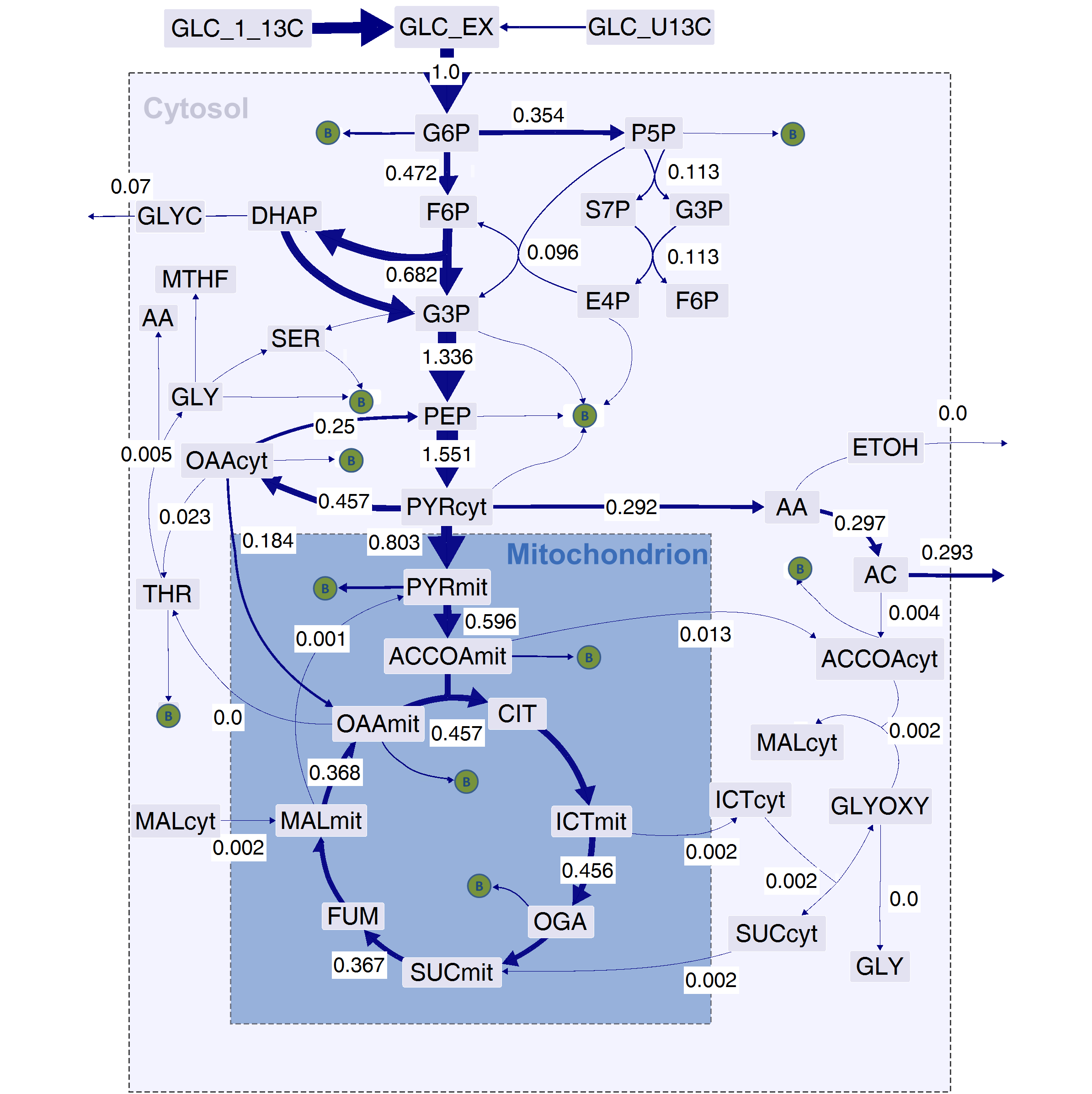


Additional file 1: Figure S9. Simulated flux distributions of K. marxianus CBS 2080 during exponential growth at 30 °C The boxed numbers next to reaction arrows represent the flux values, which were normalized to the glucose uptake rate. Arrow thickness was scaled to the flux value for enhanced visualization.


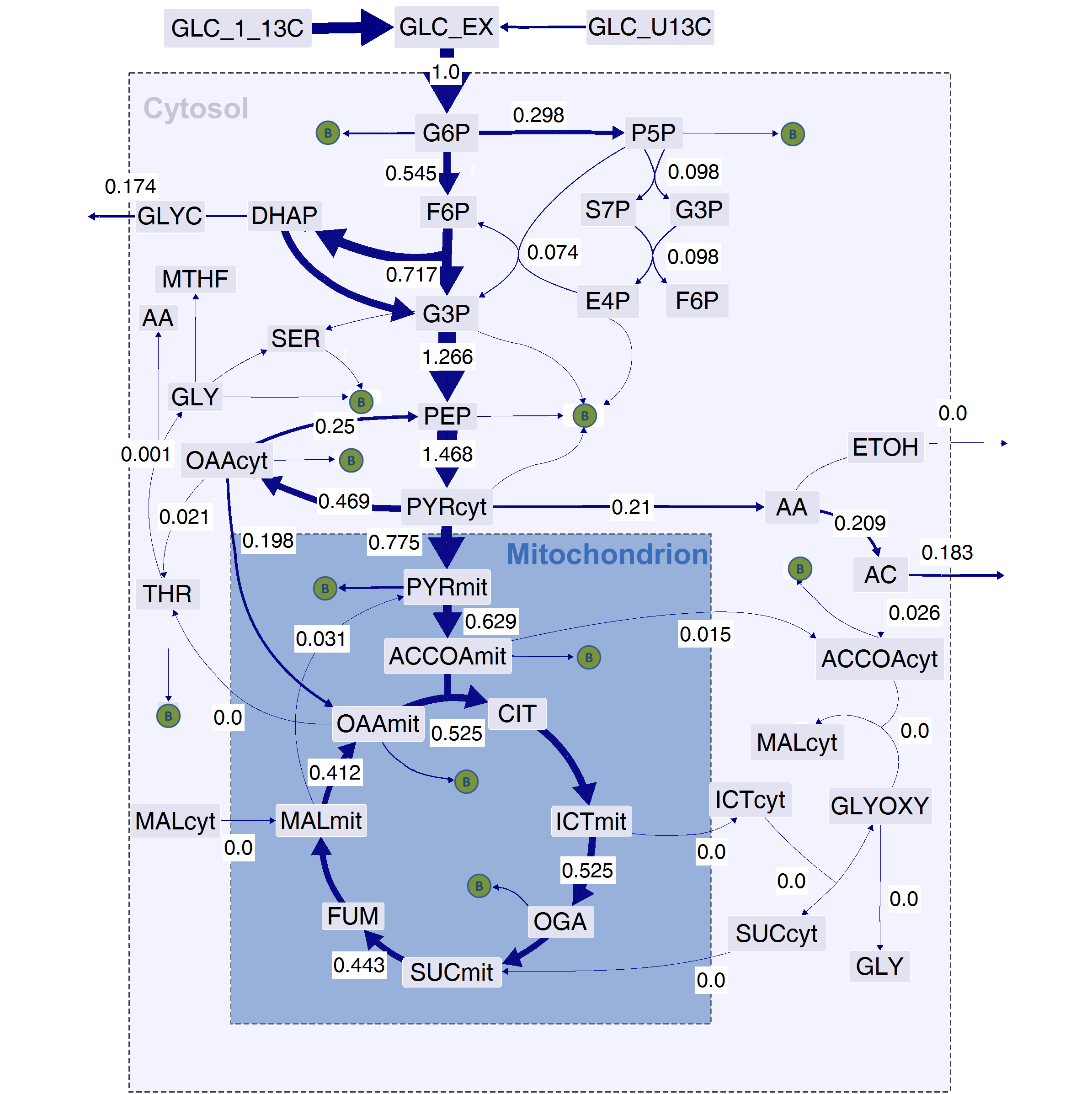


Additional file 1: Figure S10. Simulated flux distributions of K. marxianus CBS 2080 during exponential growth at 37 °C The boxed numbers next to reaction arrows represent the flux values, which were normalized to the glucose uptake rate. Arrow thickness was scaled to the flux value for enhanced visualization.


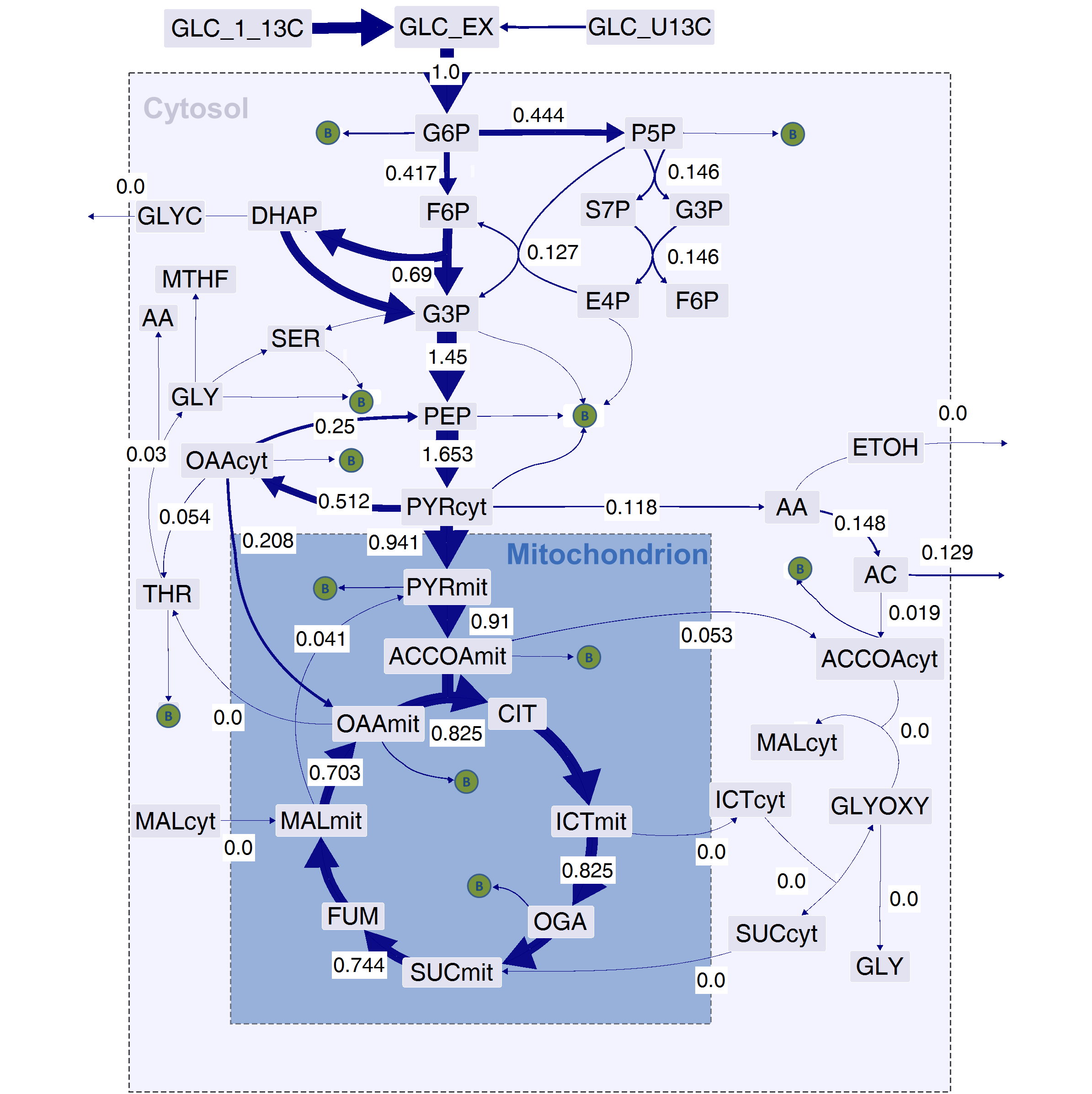


Additional file 1: Figure S11. Simulated flux distributions of K. marxianus CBS 2080 during exponential growth at 40 °C The boxed numbers next to reaction arrows represent the flux values, which were normalized to the glucose uptake rate. Arrow thickness was scaled to the flux value for enhanced visualization.


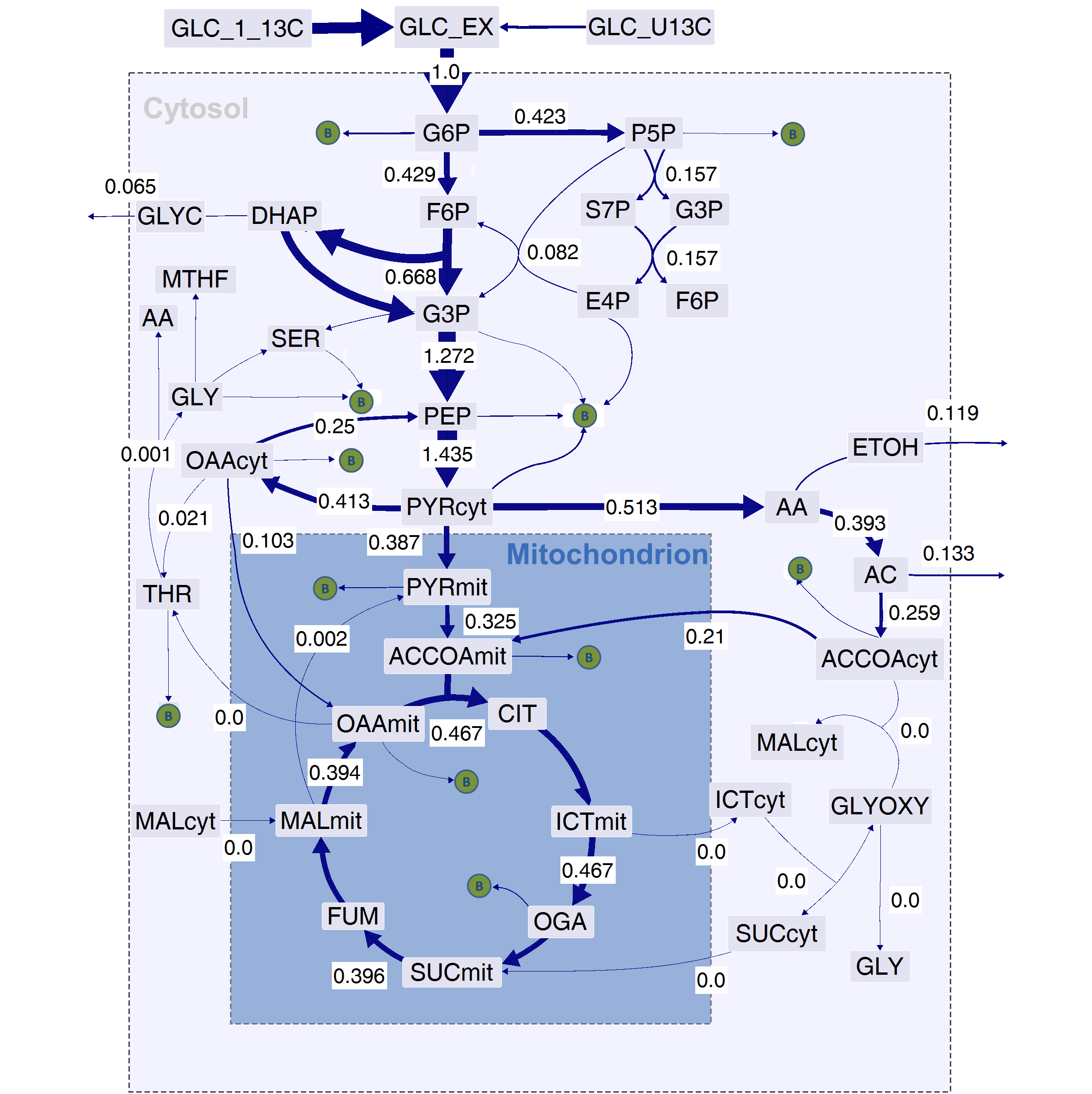


Additional file 1: Figure S12. Simulated flux distributions of K. marxianus CBS 2080 during exponential growth at 45 °C The boxed numbers next to reaction arrows represent the flux values, which were normalized to the glucose uptake rate. Arrow thickness was scaled to the flux value for enhanced visualization.


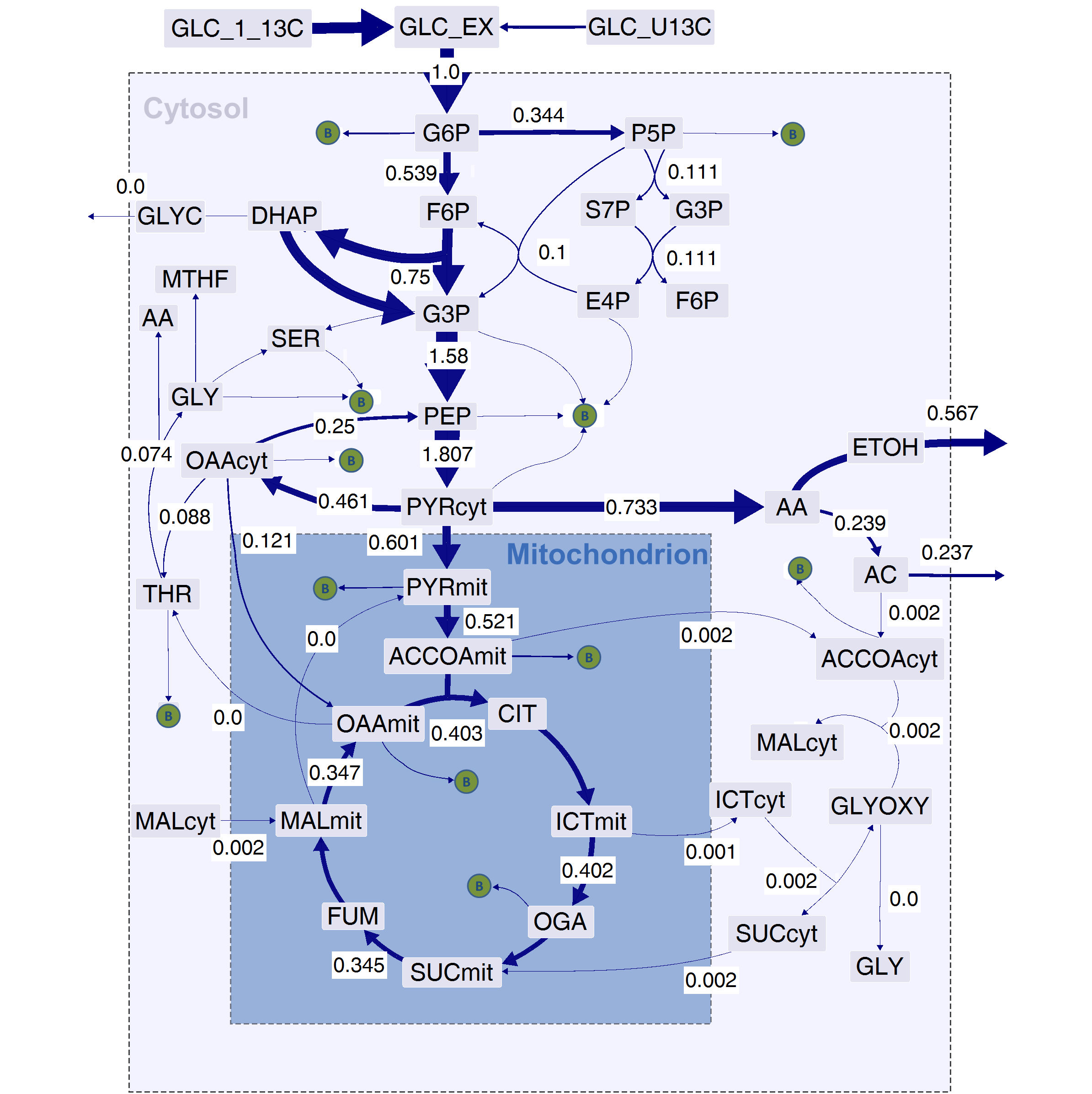


Additional file 1: Figure S13. Simulated flux distributions of K. marxianus CBS 2080 during exponential growth at 47 °C The boxed numbers next to reaction arrows represent the flux values, which were normalized to the glucose uptake rate. Arrow thickness was scaled to the flux value for enhanced visualization.


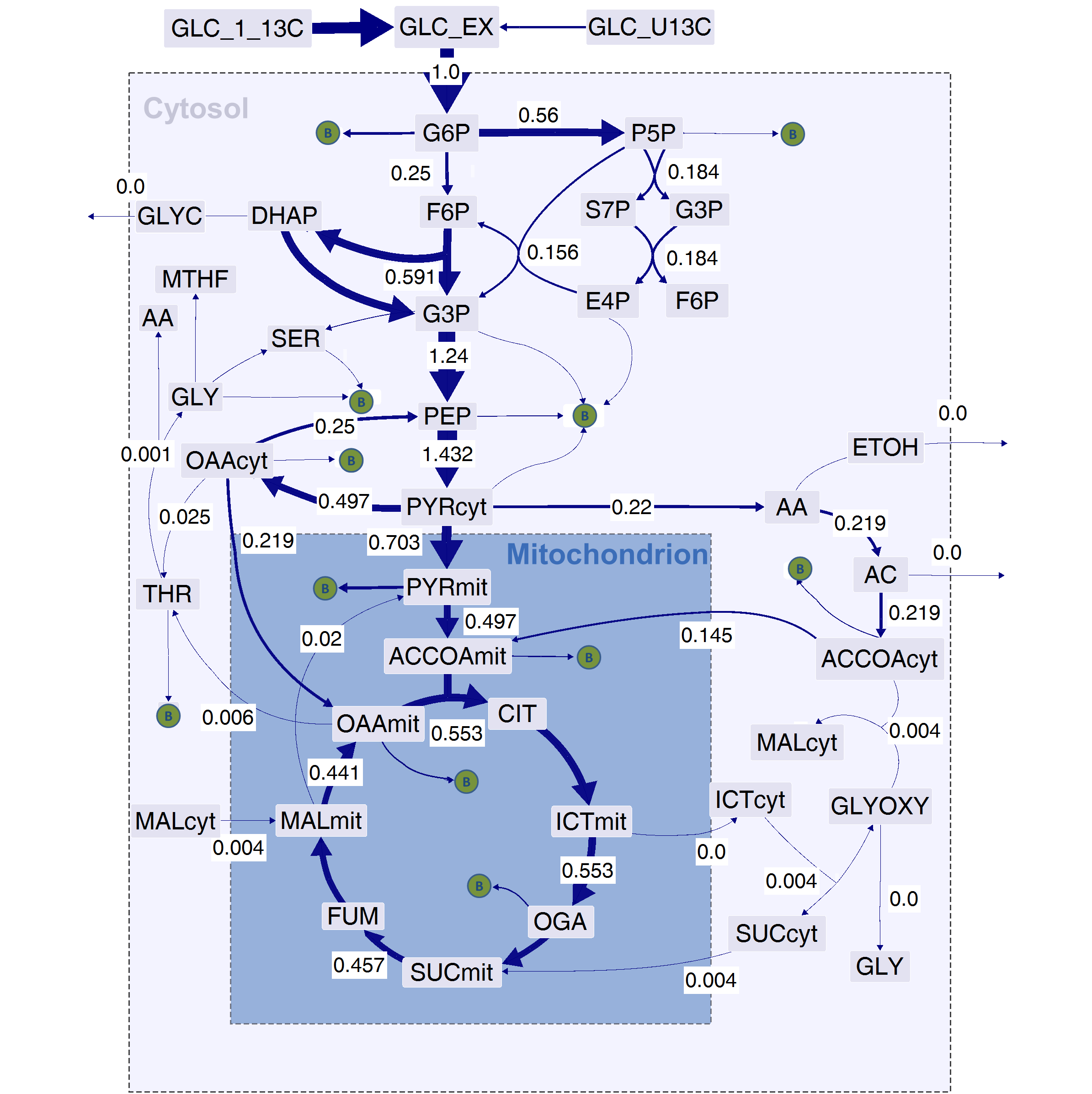


Additional file 1: Figure S14. Simulated flux distributions of O. polymorpha KCTC 17233 during exponential growth at 30 °C The boxed numbers next to reaction arrows represent the flux values, which were normalized to the glucose uptake rate. Arrow thickness was scaled to the flux value for enhanced visualization.


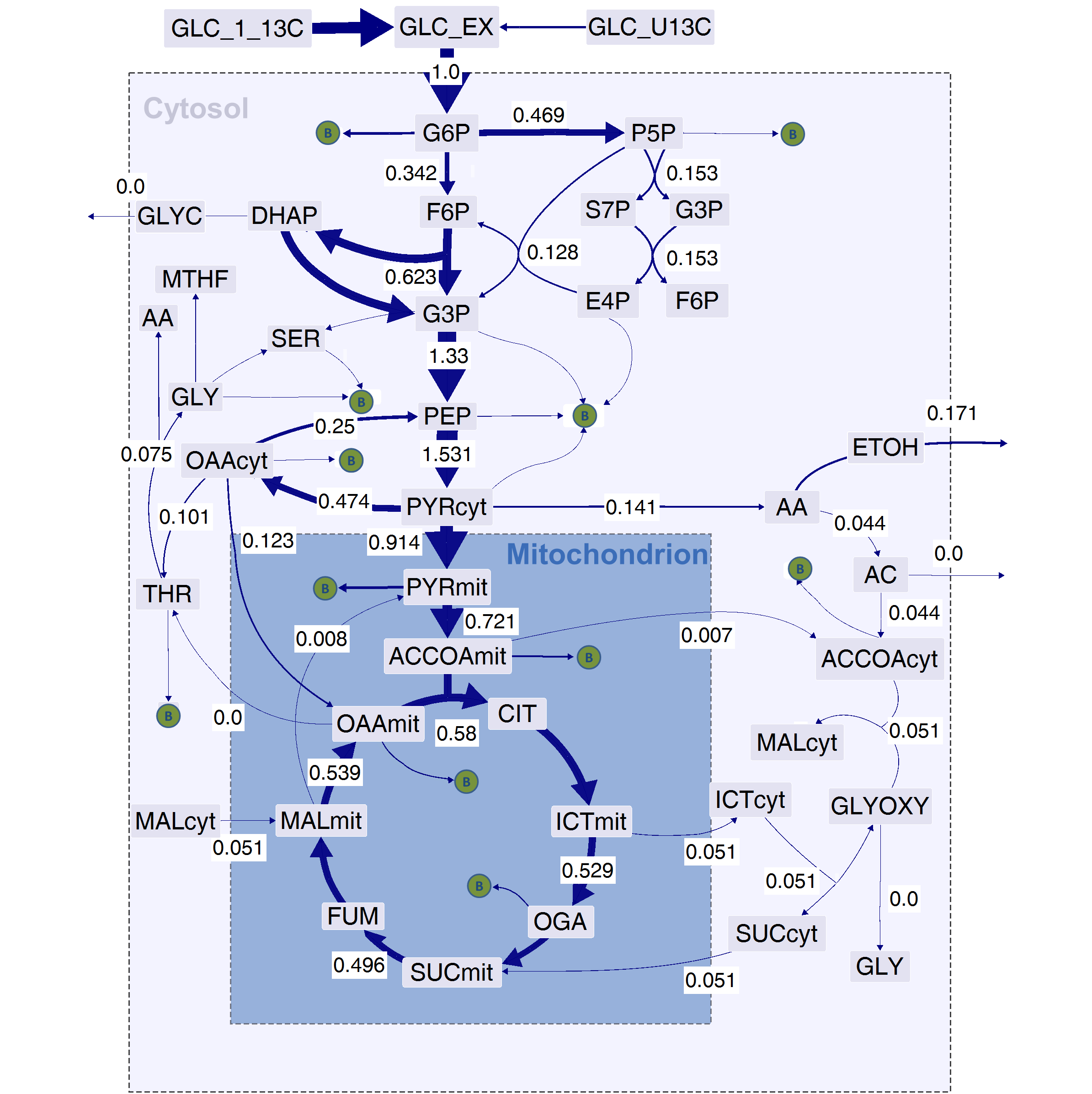


Additional file 1: Figure S15. Simulated flux distributions of O. polymorpha KCTC 17233 during exponential growth at 40 °C The boxed numbers next to reaction arrows represent the flux values, which were normalized to the glucose uptake rate. Arrow thickness was scaled to the flux value for enhanced visualization.


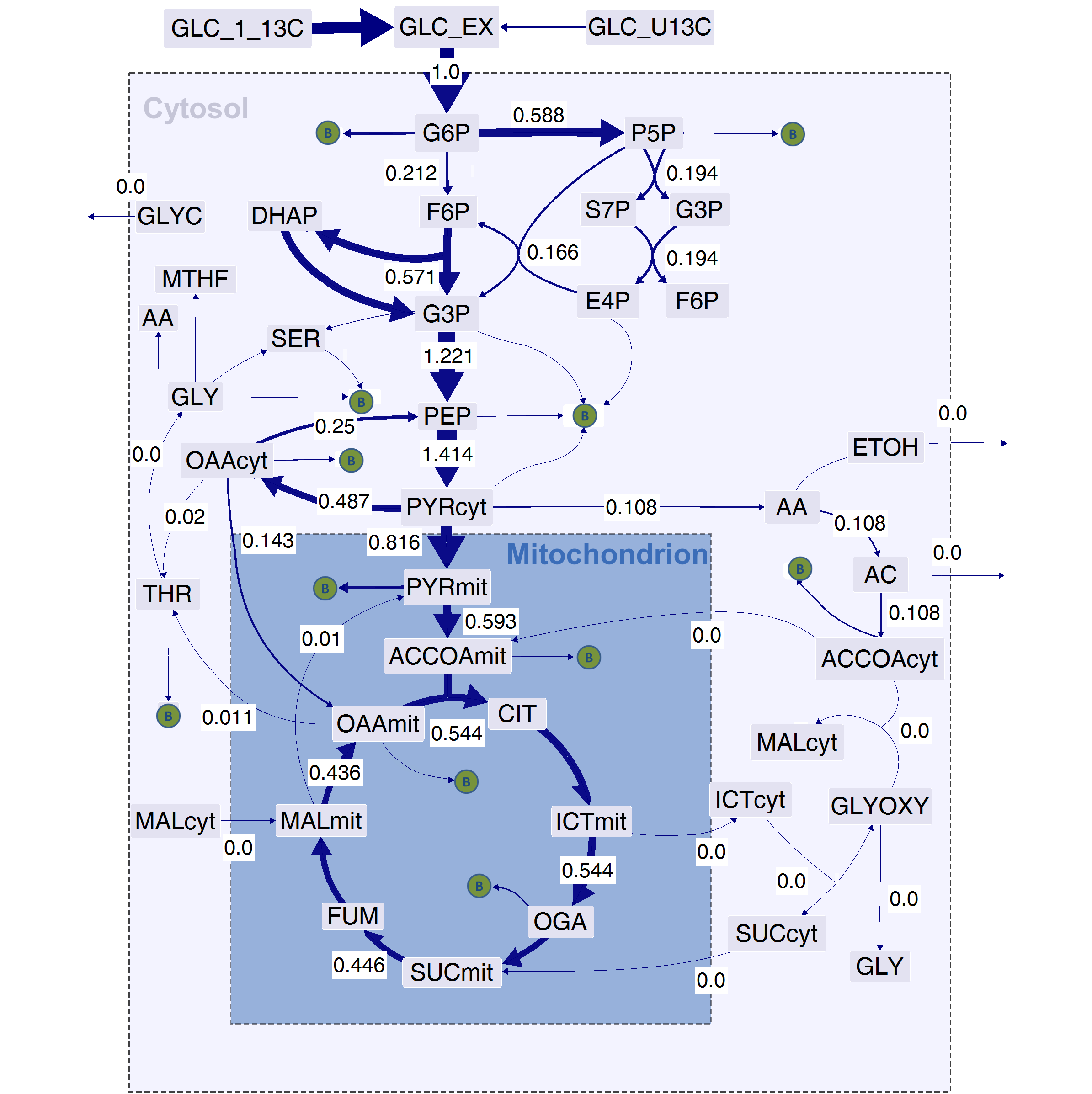


Additional file 1: Figure S16. Simulated flux distributions of O. polymorpha KCTC 17233 during exponential growth at 45 °C The boxed numbers next to reaction arrows represent the flux values, which were normalized to the glucose uptake rate. Arrow thickness was scaled to the flux value for enhanced visualization.


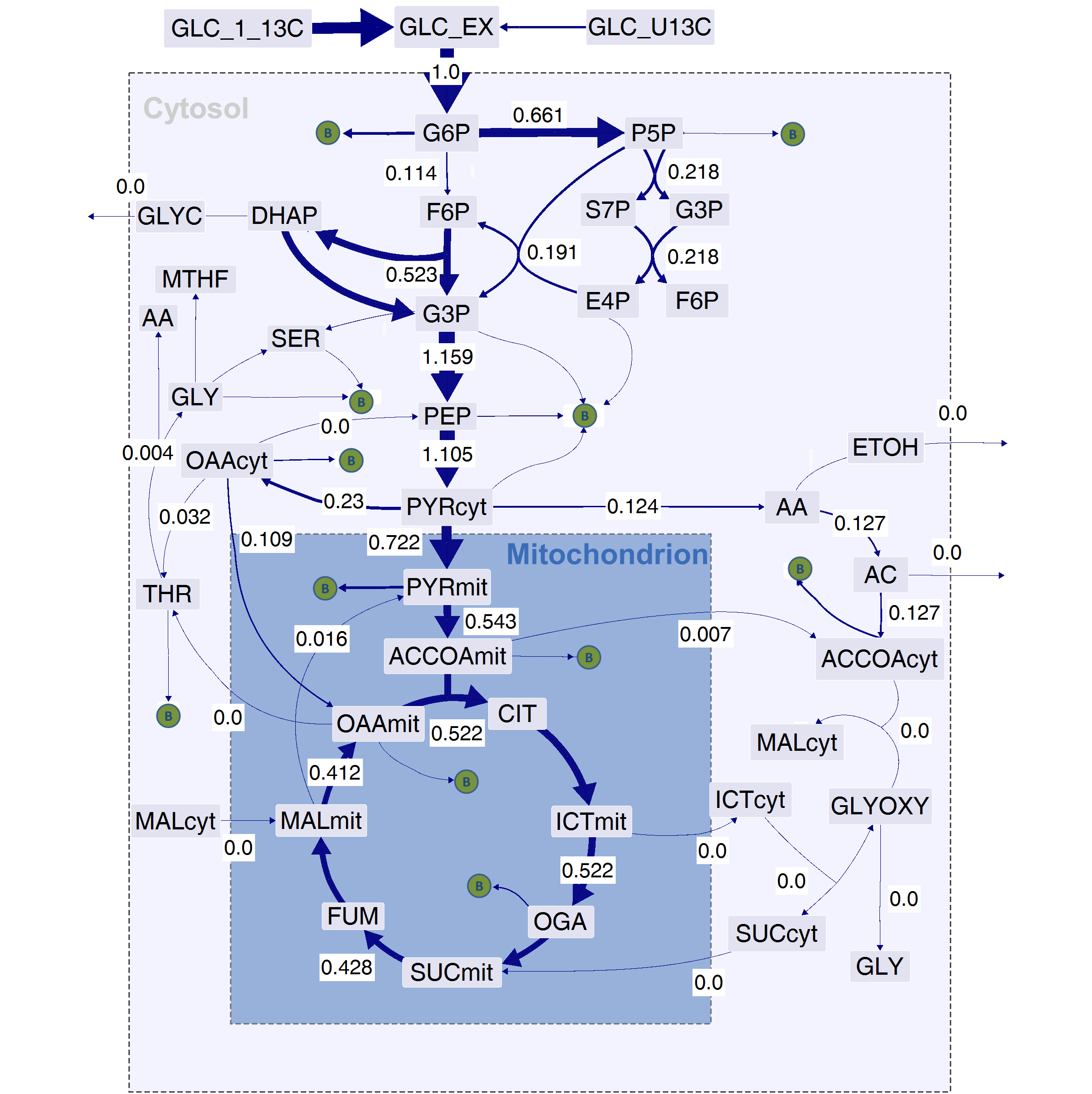


Additional file 1: Figure S17. Simulated flux distributions of O. polymorpha KCTC 17233 during exponential growth at 47 °C The boxed numbers next to reaction arrows represent the flux values, which were normalized to the glucose uptake rate. Arrow thickness was scaled to the flux value for enhanced visualization.


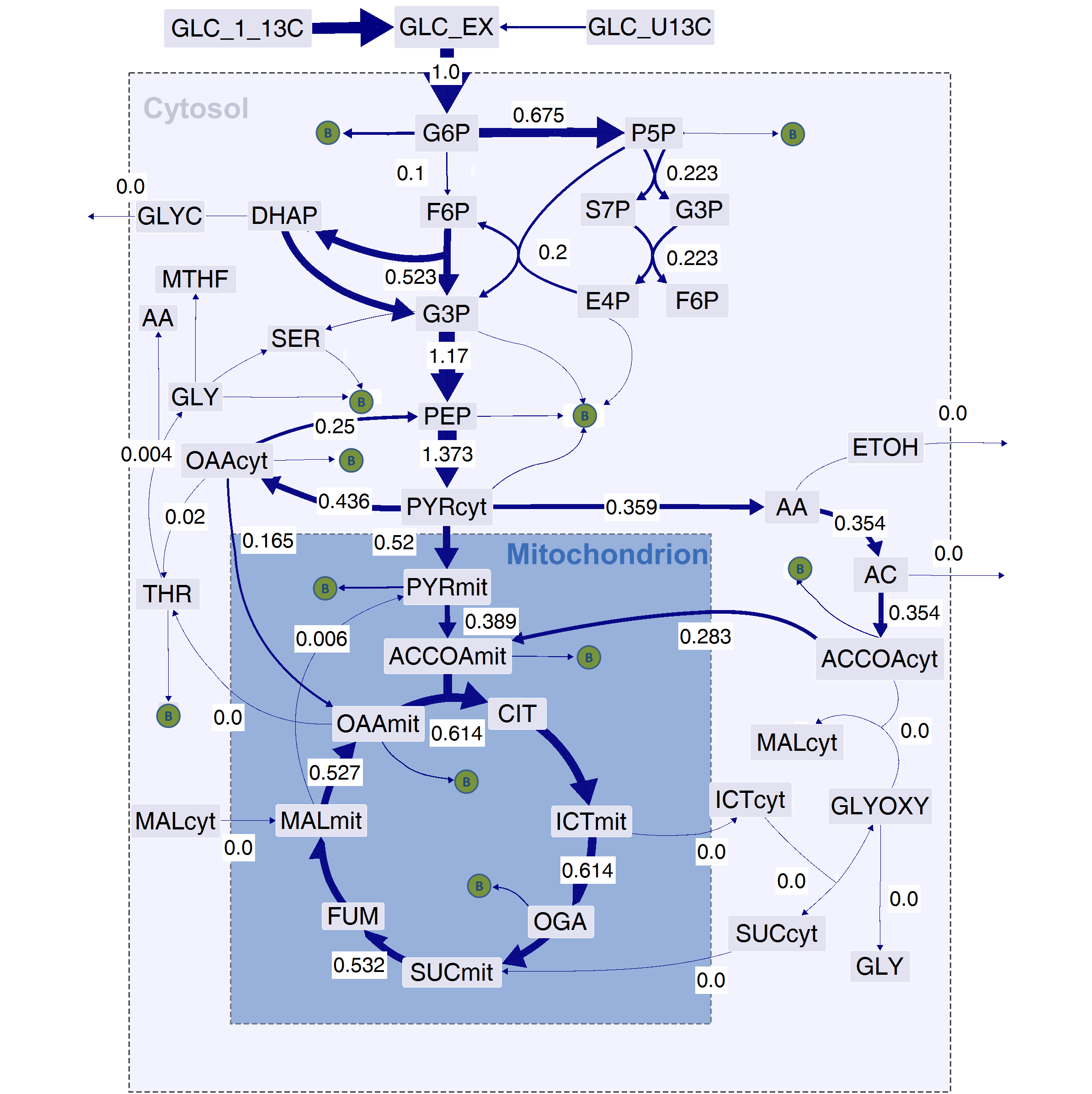


Additional file 1: Figure S18. Simulated flux distributions of O. polymorpha CLIB 421 during exponential growth at 45 °C The boxed numbers next to reaction arrows represent the flux values, which were normalized to the glucose uptake rate. Arrow thickness was scaled to the flux value for enhanced visualization.


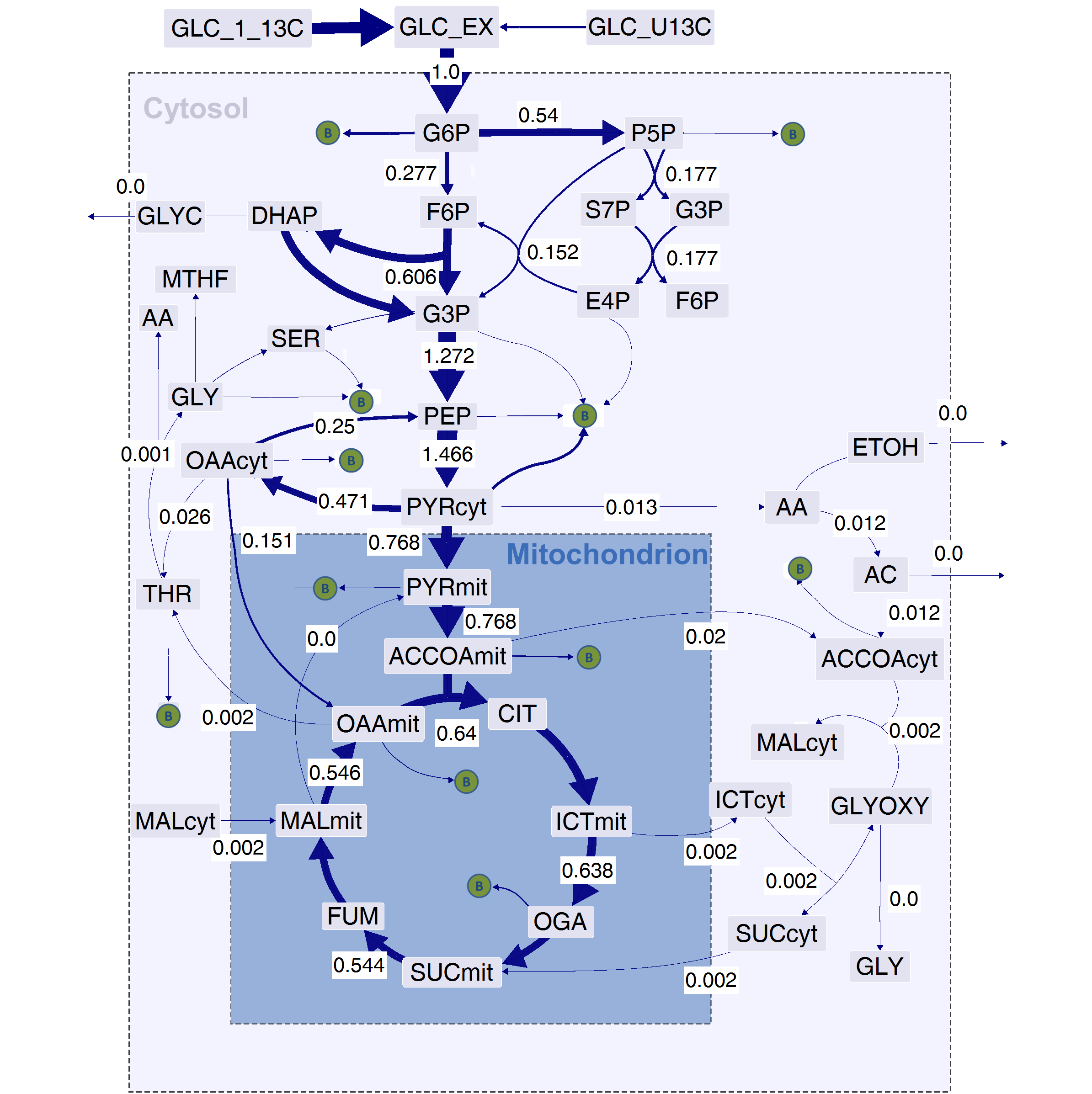


Additional file 1: Figure S19. Simulated flux distributions of O. polymorpha CLIB 421 during exponential growth at 47 °C The boxed numbers next to reaction arrows represent the flux values, which were normalized to the glucose uptake rate. Arrow thickness was scaled to the flux value for enhanced visualization.


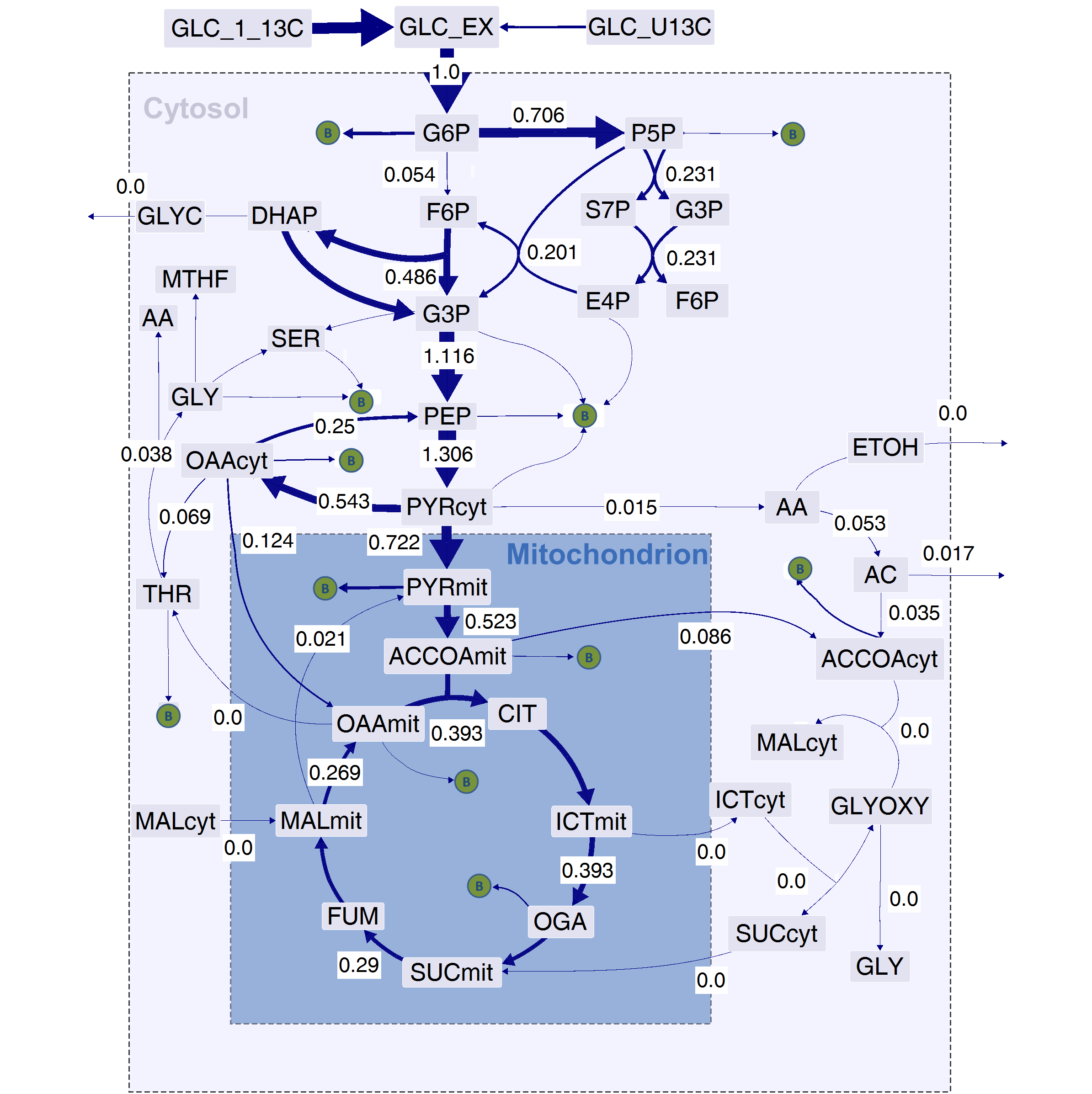


Additional file 1: Figure S20. Simulated flux distributions of O. polymorpha NCYC 496 during exponential growth at 30 °C The boxed numbers next to reaction arrows represent the flux values, which were normalized to the glucose uptake rate. Arrow thickness was scaled to the flux value for enhanced visualization.


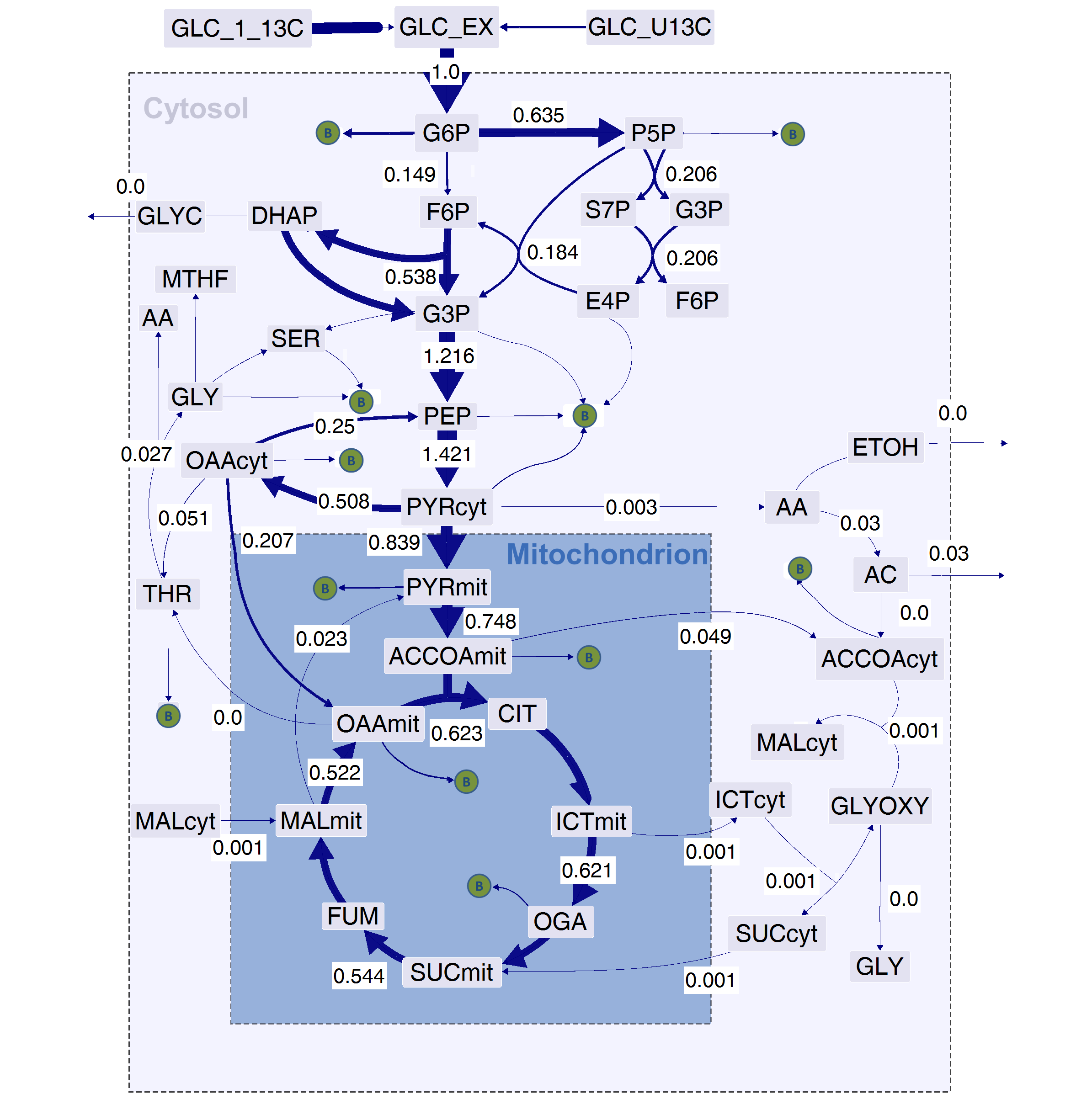


Additional file 1: Figure S21. Simulated flux distributions of O. polymorpha NCYC 496 during exponential growth at 37 °C The boxed numbers next to reaction arrows represent the flux values, which were normalized to the glucose uptake rate. Arrow thickness was scaled to the flux value for enhanced visualization.


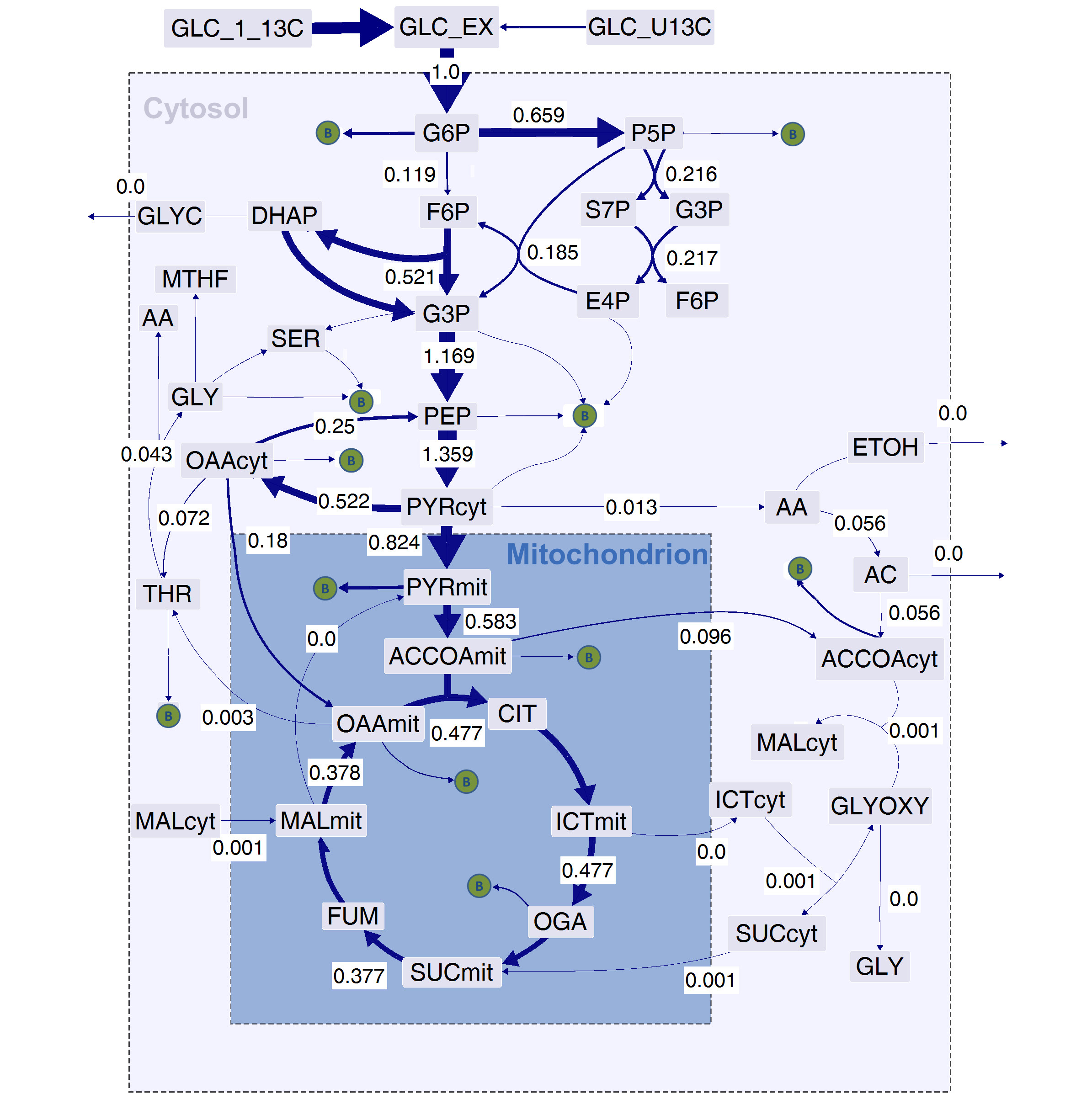


Additional file 1: Figure S22. Simulated flux distributions of O. polymorpha NCYC 496 during exponential growth at 40 °C The boxed numbers next to reaction arrows represent the flux values, which were normalized to the glucose uptake rate. Arrow thickness was scaled to the flux value for enhanced visualization.


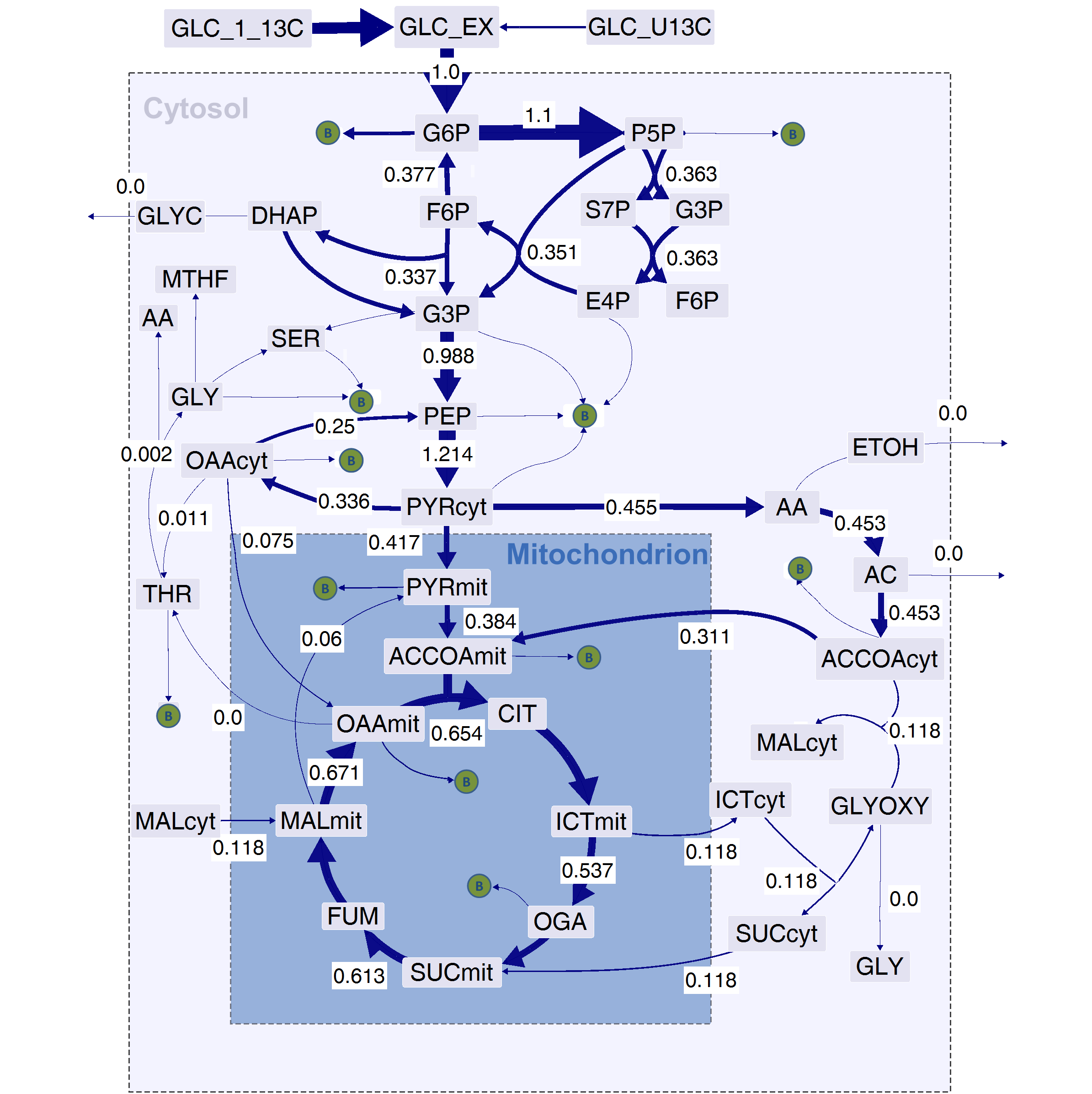


Additional file 1: Figure S23. Simulated flux distributions of O. polymorpha NCYC 496 during exponential growth at 45 °C The boxed numbers next to reaction arrows represent the flux values, which were normalized to the glucose uptake rate. Arrow thickness was scaled to the flux value for enhanced visualization.


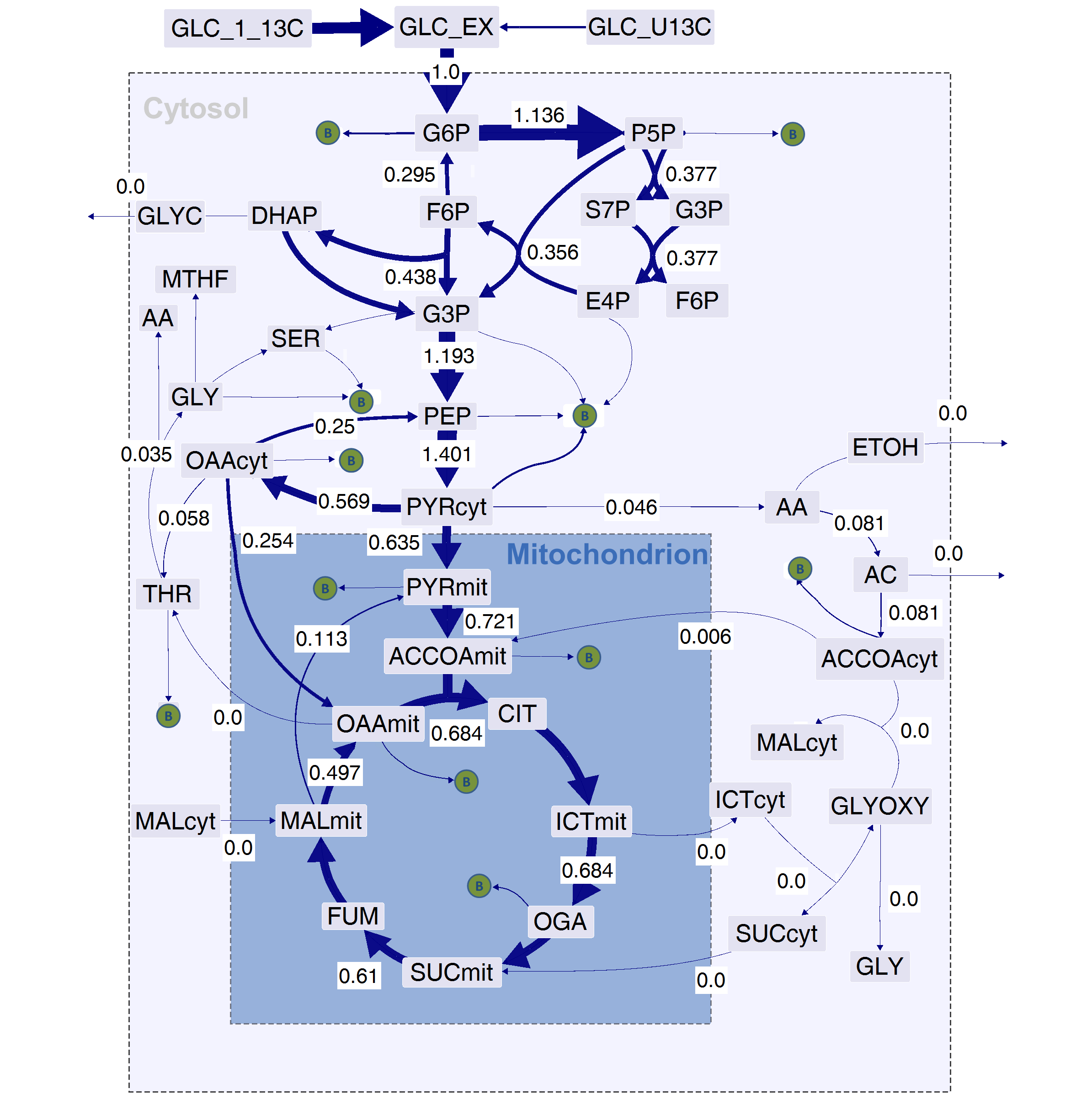


Additional file 1: Figure S24. Simulated flux distributions of O. polymorpha NCYC 496 during exponential growth at 47 °C The boxed numbers next to reaction arrows represent the flux values, which were normalized to the glucose uptake rate. Arrow thickness was scaled to the flux value for enhanced visualization.


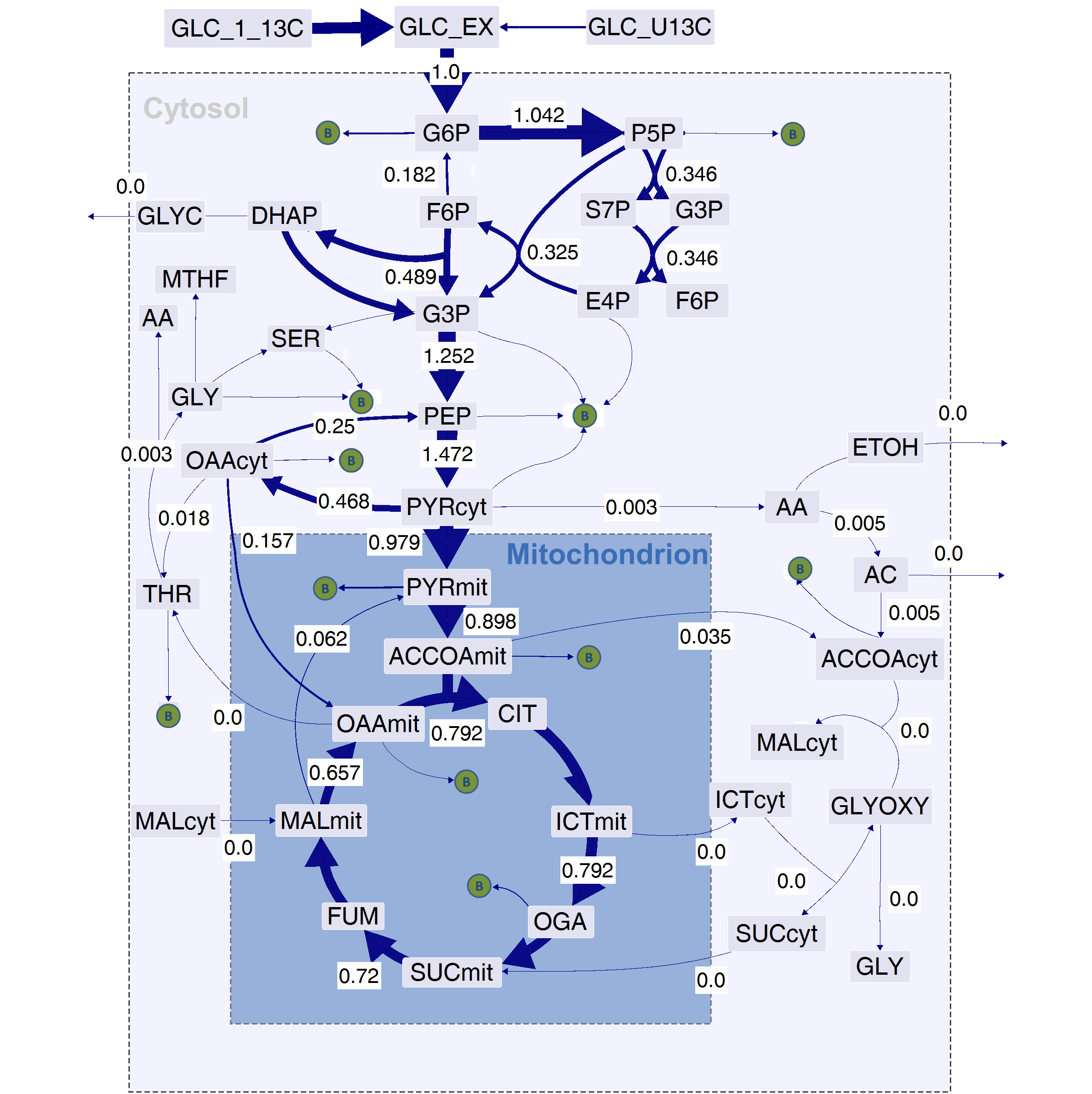


Additional file 1: Figure S25. Simulated flux distributions of O. polymorpha NCYC 496 during exponential growth at 49 °C The boxed numbers next to reaction arrows represent the flux values, which were normalized to the glucose uptake rate. Arrow thickness was scaled to the flux value for enhanced visualization.
