# Supplementary material for: Elevated temperatures do not trigger a conserved metabolic network response among thermotolerant yeasts
Source: BMC Microbiol. 2019 May 17;19:100. doi: 10.1186/s12866-019-1453-3 (PMC6525440; doi:10.1186/s12866-019-1453-3)
Supplement: Supplementary file 2 — Table S1. Physiological rates of K. marxianus strains from batch experiments on glucose at different temperatures. Depending on the strain, the cultivation temperature was varied between 30 °C and 49 °C in different experiments. Table S2. Physiological rates of O. polymorpha strains from batch experiments on glucose at different temperatures. Depending on the strain, the cultivation temperature was varied between 30 °C and 49 °C in different experiments. (DOCX 26 kb) [file 12866_2019_1453_MOESM2_ESM.docx]

Additional file 2: Table S1. Physiological rates of K. marxianus strains from batch experiments on glucose at different temperatures. Depending on the strain, the cultivation temperature was varied between 30 °C and 49 °C in different experiments. µ - specific growth rate; r_glucose_ – specific glucose uptake rate; r_O2_ – specific oxygen uptake rate; r_glycerol_ – specific glycerol production rate; r_acetate_ – specific acetate production rate; r_ethanol_ – specific ethanol production rate; r_CO2_ – specific CO_2_ production rate.

|  | *K. marxianus* CBS 712 | | | | *K. marxianus* ATCC 748 | | | | *K. marxianus* CBS 2080 | | | | |
| --- | --- | --- | --- | --- | --- | --- | --- | --- | --- | --- | --- | --- | --- |
|  | 30 °C | 37 °C | 40 °C | 45 °C | 30 °C | 37 °C | 40 °C | 47 °C | 30 °C | 37 °C | 40 °C | 45 °C | 47 °C |
| µ [h^-1^] | 0.48  ±0.01 | 0.68  ±0.01 | 0.71  ±0.02 | 0.40  ±0.01 | 0.63  ±0.02 | 0.79  ±0.01 | 0.94  ±0.02 | 0.17  ±0.00 | 0.39  ±0.01 | 0.69  ±0.01 | 0.93  ±0.01 | 0.53  ±0.01 | 0.28  ±0.01 |
| r_glucose_ [mmol/g_CDW_/h] | 5.65  ±0.46 | 9.82  ±0.69 | 10.48  ±0.47 | 4.02  ±0.27 | 6.43  ±0.76 | 10.28  ±0.56 | 10.36  ±1.47 | 9.82  ±1.03 | 5.06  ±0.54 | 9.16  ±1.21 | 11.48  ±1.24 | 7.48  ±0.35 | 6.06  ±0.7 |
| r_O2_ [mmol/g_CDW_/h] | 12.94  ±0.52 | 14.78  ±1.45 | 15.49  ±3.03 | 7.17  ±1.51 | 12.45  ±0.72 | 11.66  ±2.3 | 25.38  ±2.41 | 5.67  ±0.2 | 8.25  ±0.28 | 17.63  ±2.11 | 41.72  ±6.05 | 11.37  ±1.44 | 9.08  ±0.87 |
| r_glycerol_ [mmol/g_CDW_/h] | 0.56  ±0.04 | 1  ±0.14 | 1.59  ±0.08 | 0.49  ±0.04 | 0.55  ±0.07 | 0.93  ±0.05 | 0  ±0 | 1.12  ±0.11 | 0.31  ±0.03 | 0.76  ±0.09 | 0  ±0 | 0.23  ±0.23 | 0  ±0 |
| r_acetate_ [mmol/g_CDW_/h] | 0.7  ±0.05 | 2.31  ±0.18 | 0.8  ±0.81 | 0.16  ±0.16 | 0.52  ±0.07 | 0.52  ±0.03 | 0.63  ±0.06 | 0.48  ±0.06 | 1.79  ±0.17 | 1.84  ±0.19 | 1.75  ±0.18 | 0.91  ±0.04 | 1.74  ±0.22 |
| r_ethanol_ [mmol/g_CDW_/h] | 0  ±0 | 1.49  ±1.49 | 1.59  ±1.59 | 0  ±0 | 0  ±0 | 1.58  ±0.81 | 0  ±0 | 13.86  ±1.33 | 0  ±0 | 0  ±0 | 0  ±0 | 0.65  ±0.69 | 3.63  ±0.34 |
| r_CO2_ [mmol/g_CDW_/h] | 11.48  ±0.35 | 13.55  ±1.6 | 15.01  ±0.5 | 7.11  ±0.39 | 12.98  ±0.83 | 12.65  ±0.55 | 28.05  ±1.63 | 13.23  ±0.98 | 8.46  ±0.3 | 16.77  ±0.83 | 52.42  ±5.01 | 13.58  ±0.5 | 10.22  ±0.99 |

Additional file 2: Table S2. Physiological rates of O. polymorpha strains from batch experiments on glucose at different temperatures. Depending on the strain, the cultivation temperature was varied between 30 °C and 49 °C in different experiments. µ - specific growth rate; r_glucose_ – specific glucose uptake rate; r_O2_ – specific oxygen uptake rate; r_glycerol_ – specific glycerol production rate; r_acetate_ – specific acetate production rate; r_ethanol_ – specific ethanol production rate; r_CO2_ – specific CO_2_ production rate.

|  | *O. polymorpha* KCTC 17233 | | | | | *O. polymorpha* CLIB 421 | | *O. polymorpha* NCYC 495 | | | | | |
| --- | --- | --- | --- | --- | --- | --- | --- | --- | --- | --- | --- | --- | --- |
|  | 30 °C | 37 °C | 40 °C | 45 °C | 47 °C | 45 °C | 47 °C | 30 °C | 37 °C | 40 °C | 45 °C | 47 °C | 49 °C |
| µ [h^-1^] | 0.39  ±0 | 0.42  ±0.01 | 0.44  ±0.01 | 0.59  ±0 | 0.44  ±0.02 | 0.52  ±0 | 0.39  ±0 | 0.44  ±0.02 | 0.46  ±0.03 | 0.48  ±0.01 | 0.36  ±0 | 0.3  ±0.01 | 0.2  ±0.01 |
| r_glucose_ [mmol/g_CDW_/h] | 4.1  ±0.05 | 4.44  ±0.24 | 4.46  ±0.46 | 5.68  ±0.17 | 4.24  ±0.48 | 4.87  ±0.47 | 4.52  ±0.23 | 4.25  ±0.41 | 4.71  ±0.71 | 4.6  ±0.34 | 4.25  ±0.23 | 3.44  ±0.1 | 3.04  ±0.44 |
| r_O2_ [mmol/g_CDW_/h] | 7.97  ±0.17 | 7.79  ±1.56 | 8.23  ±1.12 | 11.71  ±1.01 | 8.8  ±2.58 | 10.28  ±1.22 | 11.69  ±2.51 | 6.17  ±2.42 | 9.55  ±0.66 | 9.64  ±1.96 | 9.62  ±1.31 | 9.08  ±0.44 | 9.38  ±2.34 |
| r_glycerol_ [mmol/g_CDW_/h] | 0  ±0 | 0  ±0 | 0  ±0 | 0  ±0 | 0  ±0 | 0  ±0 | 0  ±0 | 0  ±0 | 0  ±0 | 0  ±0 | 0  ±0 | 0  ±0 | 0  ±0 |
| r_acetate_ [mmol/g_CDW_/h] | 0  ±0 | 2.25  ±0.16 | 0.92  ±0.1 | 0  ±0 | 0  ±0 | 0  ±0 | 0  ±0 | 0.07  ±0 | 0.17  ±0.04 | 0  ±0 | 0  ±0 | 0  ±0 | 0  ±0 |
| r_ethanol_ [mmol/g_CDW_/h] | 0  ±0 | 0  ±0 | 0  ±0 | 0  ±0 | 0  ±0 | 0  ±0 | 0  ±0 | 0  ±0 | 0  ±0 | 0  ±0 | 0  ±0 | 0  ±0 | 0  ±0 |
| r_CO2_ [mmol/g_CDW_/h] | 7.24  ±0.59 | 7.62  ±0.58 | 7.82  ±0.45 | 12.74  ±0.92 | 9.64  ±0.55 | 10.01  ±0.4 | 11.97  ±0.5 | 6.61  ±2.22 | 8.52  ±1.07 | 9.66  ±0.45 | 9.75  ±0.2 | 9.74  ±0.18 | 7.8  ±1.33 |
